# Study of the Extraction Process, Chemical Composition, and Biological Activities of Total Flavonoids from Vine Tea Leaves

**DOI:** 10.3390/molecules31203685

**Published:** 2026-10-14

**Authors:** Lihui Zhang, Qiuya Wu, Xiao-Qun Yang, Huijing Ren, Lihong Yang, Meizhong Hu, Dongyu Pan, Shuang Yang, Dingfeng Su, Wenrui Yue, Qin Liu, Zhiqiang Yan, Ting-Chi Wen, Pheaktra Phal

**Affiliations:** 1 School of Pharmaceutical Sciences, Tongren Polytechnic University, No. 2 Ziyou Road, Bijiang District, Tongren 554300, China; lihui8852032@163.com (L.Z.); wqy3218240887@163.com (Q.W.); yangxiaoqun95@163.com (X.-Q.Y.); rhj828823@163.com (H.R.); ylhhaha2025@163.com (L.Y.); 20080083@trzy.edu.cn (M.H.); 16683955209@163.com (D.P.); m1988517566@163.com (S.Y.); 2 Guizhou Jiangkou Fanjingshan Medical Technology Group Co., Ltd., Tongren 554400, China; sdf9992566@163.com (D.S.); 19521465716@163.com (W.Y.); lqsbw999@163.com (Q.L.); 3 Chongqing Academy of Animal Sciences, Chongqing 402460, China; 18883359161@163.com; 4 Engineering Research Center of Southwest Bio-Pharmaceutical Resources, Ministry of Education, Guizhou University, Guiyang 550025, China; tingchiwen@yahoo.com

**Keywords:** vine tea leaves, total flavonoids, extraction process, chemical composition, biological activities

## Abstract

This study investigated ethanol-based extraction of total flavonoids from vine tea (*Ampelopsis grossedentata*) leaves and characterized their chemical composition and in vitro biological activities. Ultrasound-assisted and heat-reflux extraction conditions were first screened by single-factor experiments and then evaluated using unreplicated L9(3^3^) orthogonal designs. For heat reflux extraction, the solid-to-liquid ratio had the greatest effect on flavonoid yield among the factors evaluated. The highest observed yield (58.20 mg rutin equivalents [RE]/g) was obtained with 70% ethanol, a 1:10 solid-to-liquid ratio, and 60 min (TCY6), representing the best-performing condition within the L9 array. TCY3 and TCY9 were not additional optima; they were retained as high-yield heat reflux comparison extracts for subsequent biological evaluation. Because the L9 runs were not independently replicated, the selected condition should be regarded as provisional, pending confirmation with replicated extraction batches. UPLC-QTOF-MS/MS profiling generated 94 metabolite annotations, comprising 43 higher-confidence assignments confirmed by accurate-mass matching and comparison with authentic reference standards and 51 tentative assignments supported by high-resolution MS/MS fragmentation. Flavones, flavonols, and flavanones accounted for 25.53%, 18.09%, and 15.96% of the annotated features, respectively. The extracts exhibited strong antioxidant activity, as demonstrated by their DPPH and ABTS radical-scavenging activities and ferric-reducing antioxidant power. In addition, they inhibited the growth of *Escherichia coli*, *Salmonella* spp., and *Staphylococcus aureus*, with the strongest antibacterial activity observed against *S. aureus*. In lipopolysaccharide (LPS)-stimulated RAW264.7 macrophages, TCY3, TCY6, and TCY9 significantly reduced malondialdehyde (MDA) levels while enhancing the activities of glutathione peroxidase (GSH-Px), superoxide dismutase (SOD), and catalase (CAT) (*p* < 0.01). Furthermore, all three extracts significantly increased the level of interleukin-10 (IL-10) while decreasing the production of the pro-inflammatory interleukin-1β (IL-1β), interleukin-6 (IL-6), and tumor necrosis factor-α (TNF-α) (*p* < 0.01). Collectively, total flavonoids extracted from vine tea leaves under the optimized conditions exhibited potent antioxidant, antibacterial, and anti-inflammatory activities, highlighting their potential as natural bioactive ingredients for functional food and pharmaceutical applications.

## 1. Introduction

Vine tea is a traditional herbal tea prepared from the processed tender shoots of *Ampelopsis grossedentata* (Hand.-Mazz.) W. T. Wang, a species belonging to the genus *Ampelopsis* in the family Vitaceae [1,2]. It has a long history of use as both a food and a medicinal plant among ethnic minority communities in southern China and is commonly known as “Mei tea,” “White tea,” or “Duanwu tea.” According to traditional Chinese medicine, vine tea is considered to possess a cooling property and a mildly sweet taste. It has traditionally been used to treat sore throat, fever associated with the common cold, icteric hepatitis, and dyspepsia [3,4]. Modern phytochemical studies have demonstrated that vine tea is rich in bioactive constituents, including flavonoids, phenolic compounds, steroids, and terpenoids [5,6]. These compounds contribute to its diverse pharmacological activities, including antimicrobial, anti-inflammatory, antioxidant, and immunomodulatory effects, as well as the regulation of glucose and lipid metabolism, hepatoprotective activity, and inhibition of tumor cell proliferation [7,8]. Owing to these beneficial biological properties, vine tea has attracted considerable attention as a natural source of bioactive compounds and has been increasingly applied as a natural preservative and functional ingredient in the food industry [9,10].

Vine tea is considered one of the plant species with the highest known flavonoid contents, accounting for more than 30% of its dry weight [11,12]. Dihydromyricetin is its principal characteristic constituent, while other major flavonoids include myricetin, quercetin, and various flavonoid glycosides [10,13]. Advanced extraction strategies have been applied to improve the extraction of bioactive constituents from vine tea. For instance, Cai X et al. [14] reported ultrasound-assisted deep eutectic solvent (DES) extraction with favorable flavonoid recovery and antioxidant activity. Nevertheless, DES systems may introduce practical considerations related to solvent formulation, downstream separation, regulatory acceptance, and solvent recovery. For industrially oriented processing, aqueous ethanol remains attractive because it is comparatively inexpensive, food-compatible, readily recoverable, and already familiar in botanical extraction. Therefore, systematic optimization of conventional ethanol-based extraction retains practical value even in the context of emerging solvent technologies.

Vine tea leaves surrounding the tender shoots are abundant, readily available, and inexpensive. However, they are commonly treated as processing by-products and disposed of through composting, incineration, or landfilling, resulting in resource wastage and environmental pollution. By-products of traditional Chinese medicinal plants are rich in bioactive constituents, such as flavonoids, polysaccharides, and essential oils, and possess various biological functions, including antioxidant, anti-inflammatory, and immunomodulatory activities [15].

Although numerous studies have investigated the phytochemical composition and biological activities of *Ampelopsis grossedentata*, important gaps remain. Previous studies have often focused on individual compounds, a single extraction technology, or a limited set of biological endpoints, while vine tea leaves generated during tender-shoot processing remain underutilized. In particular, it remains unclear how extraction method and ethanol concentration influence the recovery and composition of flavonoids and whether these compositional differences are associated with biological activities. Because ultrasound-assisted extraction and heat reflux extraction rely on different extraction mechanisms, whereas ethanol concentration alters solvent polarity and flavonoid selectivity, these factors may generate distinct flavonoid profiles with different antioxidant and anti-inflammatory effects; we therefore hypothesized that extraction conditions would affect not only flavonoid yield but also chemical composition and subsequent bioactivity.

Therefore, this study aimed to (i) evaluate key variables governing ethanol-based extraction of total flavonoids from vine tea leaves, (ii) qualitatively annotate the flavonoid-rich chemical profile by UPLC-QTOF-MS/MS, and (iii) compare the in vitro biological activities of selected high-yield heat-reflux extracts, thereby providing a basis for value-added utilization of this plant material and for subsequent quantitative, mechanistic, safety, and scale-up studies.

## 2. Results

### 2.1. Single-Factor Experiments

#### 2.1.1. Effect of the Solid-to-Liquid Ratio on the Extraction of Total Flavonoids from Vine Tea Leaves

**Figure 1:**
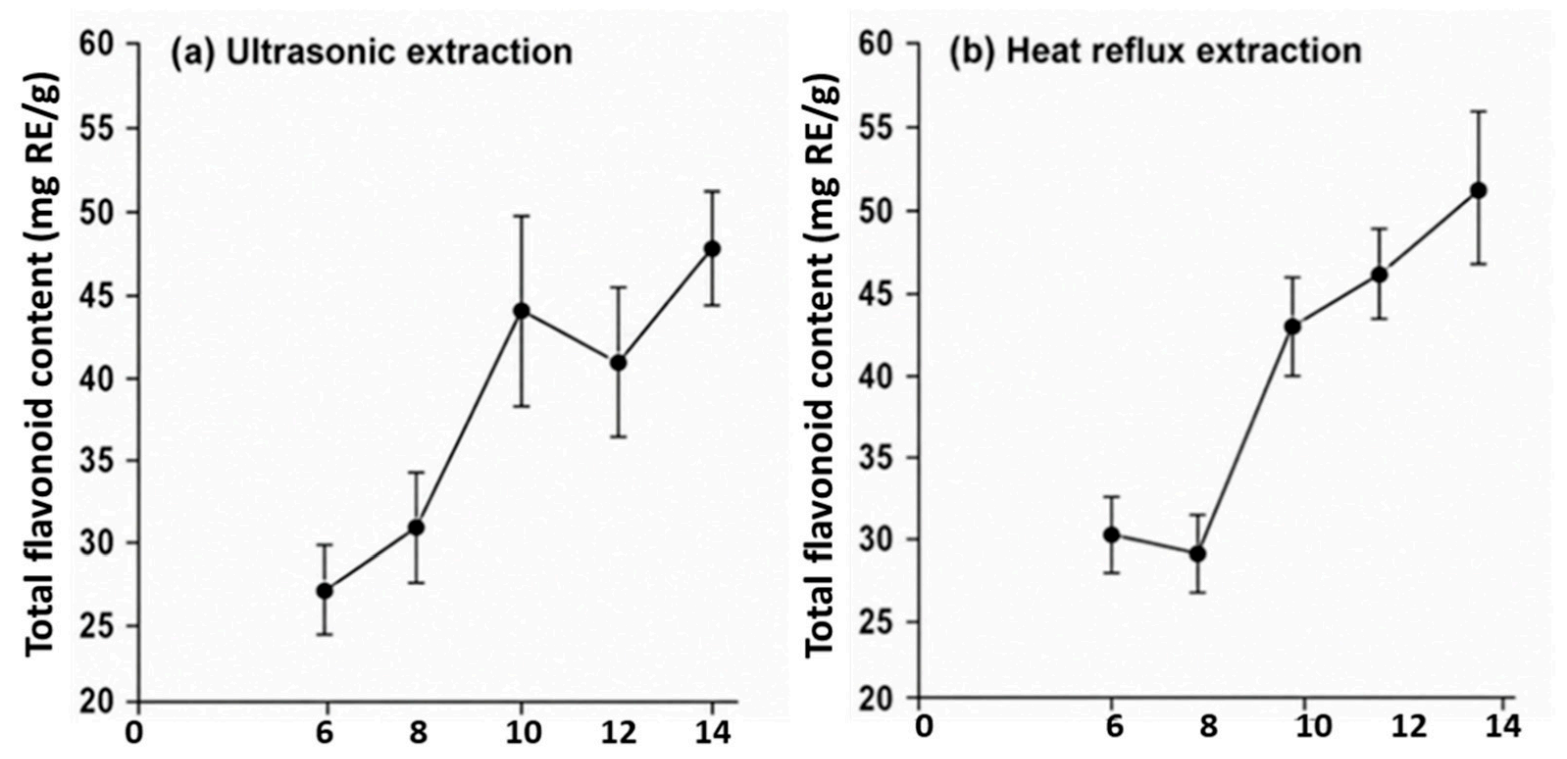
Effect of the solid-to-liquid ratio on the extraction yield of total flavonoids from vine tea leaves by (**a**) ultrasonic extraction and (**b**) heat reflux extraction. The data are expressed as mean ± standard deviation. In both extraction methods, total flavonoid content increased with increasing material-to-liquid ratio. The total flavonoid content is expressed as mg rutin equivalents (RE)/g of dry weight.

As shown in Figure 1a,b, the total flavonoid yield obtained by both ultrasonic extraction and heat reflux extraction increased as the solid-to-liquid ratio increased from 1:6 to 1:14. However, the increase in extraction yield became less pronounced when the solid-to-liquid ratio reached 1:10. Considering both extraction efficiency and solvent consumption, a solid-to-liquid ratio of 1:10 was selected for subsequent experiments.

#### 2.1.2. Effect of Ultrasonic Extraction/Heat Reflux Extraction Time on the Extraction of Total Flavonoids from Vine Tea Leaves

**Figure 2:**
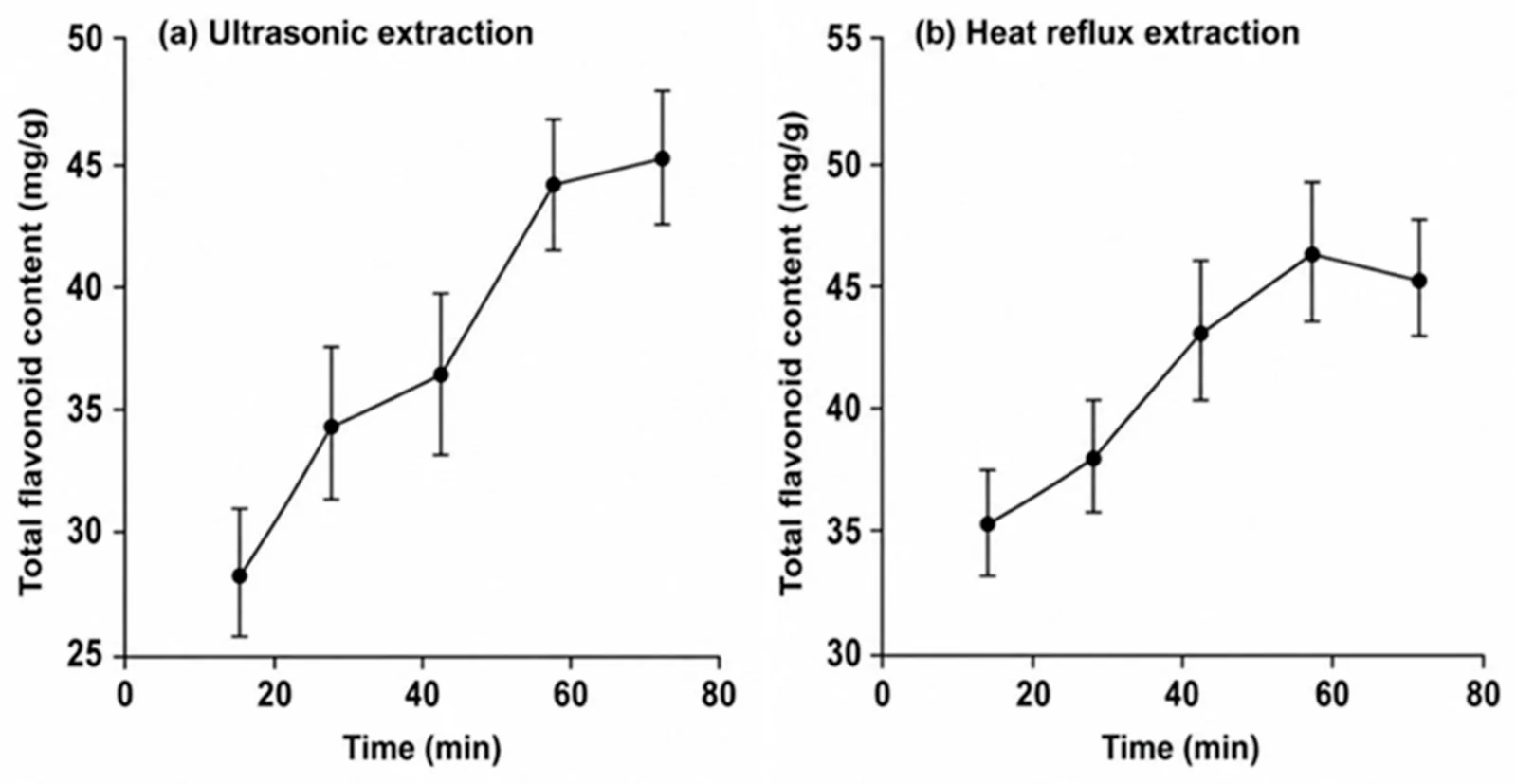
Effect of extraction time on the extraction yield of total flavonoids from vine tea leaves by (**a**) ultrasonic extraction and (**b**) heat reflux extraction. The data are expressed as mean ± standard deviation. For ultrasonic extraction, total flavonoid content increased progressively as the extraction time reached 75 min. For heat reflux extraction, total flavonoid content increased to a maximum at 60 min.

As shown in Figure 2a,b, the total flavonoid yield obtained by both ultrasonic extraction and heat reflux extraction gradually increased as the extraction time increased from 30 to 70 min. The extraction yield reached its maximum at 60 min, after which it remained relatively stable or showed a slight decline. Therefore, an extraction time of 60 min was selected for the subsequent experiments.

#### 2.1.3. Effect of Ethanol Concentration on the Extraction of Total Flavonoids from Vine Tea Leaves

**Figure 3:**
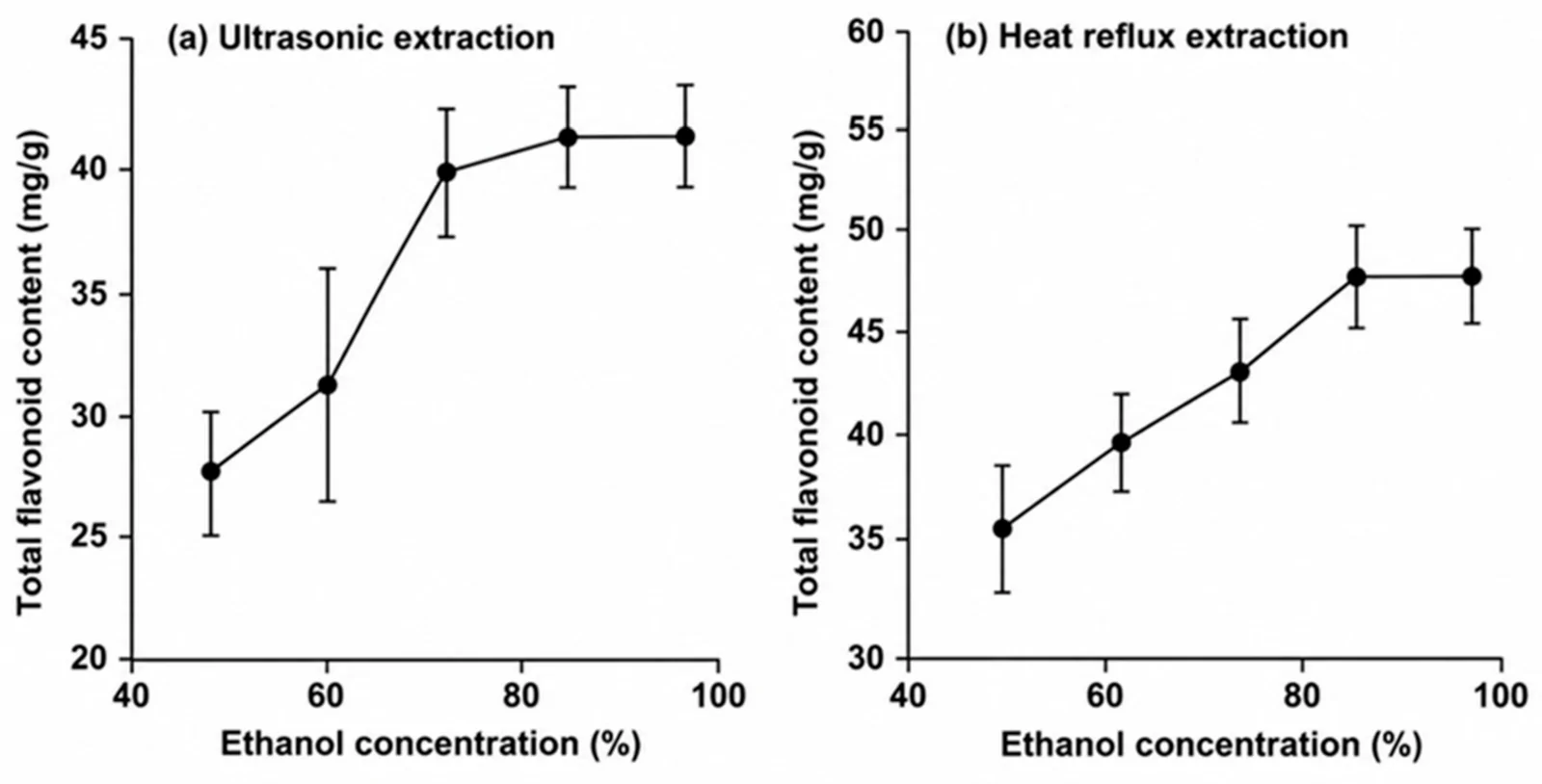
Effect of ethanol concentration on the extraction yield of total flavonoids from vine tea leaves by (**a**) ultrasonic extraction and (**b**) heat reflux extraction. The data are expressed as mean ± standard deviation. Total flavonoid content obtained by both ultrasonic extraction and heat reflux extraction increased as the ethanol concentration increased from 50% to 90%.

As shown in Figure 3a,b, flavonoid recovery depended on ethanol concentration. Intermediate aqueous-ethanol mixtures generally performed better than the most dilute or most concentrated conditions, consistent with the mixed polarity of vine tea flavonoids and the role of water in swelling the plant matrix. The ultrasonic experiment showed a maximum around 70% ethanol, whereas heat reflux favored approximately 80% in the single-factor test. Accordingly, 60%, 70%, and 80% ethanol were carried forward as the three orthogonal-design levels to test the response around this practical range.

### 2.2. Results of the Orthogonal Experiment

Based on the single-factor results, ethanol concentration (A), solid-to-liquid ratio (B), and extraction time (C) were evaluated using an L9(3^3^) orthogonal array (Table 1). Because independent replicate extractions were not performed for the L9 design, the orthogonal array data were analyzed descriptively using only level means and range (R). Accordingly, inferential statistical analyses, including ANOVA, F tests, and *p*-values, were not applied to the extraction optimization. Range analysis showed that the solid-to-liquid ratio (B) exerted the largest effect on yield for both ultrasound-assisted extraction (R = 13.93) and heat-reflux extraction (R = 13.53). For ultrasound-assisted extraction (Table 2), the factor order based on R was B > C > A, and the highest-level means predicted A2B3C3. For heat-reflux extraction (Table 3), the order was B > A > C, and the level means predicted A2B3C2; this combination corresponded to Experiment 6 and gave the highest observed yield (58.20 mg RE/g). The other heat-reflux runs yielding >50 mg RE/g, Experiments 3 and 9, were not considered additional mathematical optima; rather, they were retained as high-yield comparison extracts and designated TCY3 and TCY9, while Experiment 6 was designated TCY6. TCY3 and TCY9 are not additional mathematical optima. Because A2B3C2 was not confirmed in an independent replicate validation experiment, it is described here as the best-performing condition within the test L9 array rather than a fully validated optimum.

### 2.3. Identification Results of Chemical Constituents in Vine Tea Leaf Extracts

**Figure 4:**
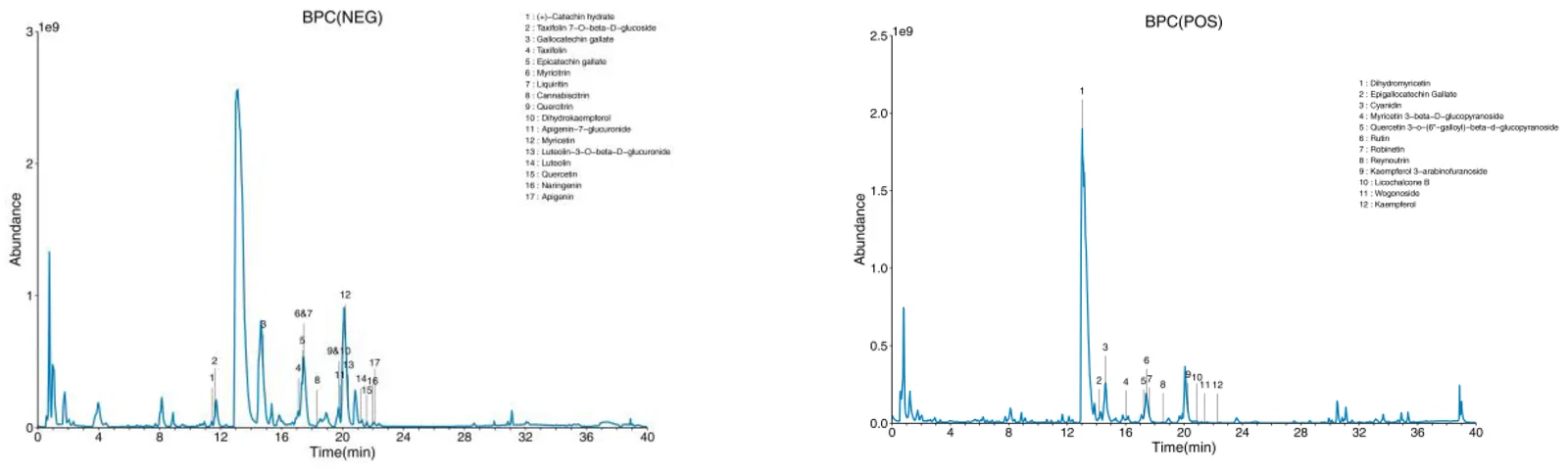
Base peak chromatograms (BPCs) of flavonoid compounds (partial) in vine tea leaf extracts obtained in ESI^+^ and ESI^−^ modes.

The identification of compounds was performed based on accurate mass measurements, MS/MS fragment ions, and isotope distribution patterns and the identification was confirmed using authentic reference standards. Qualitative analysis was conducted using the LuMet-TCM database. A total of 94 compounds were identified in vine tea leaf extracts in both positive and negative ion modes (as shown in Figure 4). Among them, 43 compounds were identified at Level 1 (accurate mass identification confirmed using authentic reference standards), including dihydromyricetin, luteolin, myricetin, quercetin, kaempferol, and others, as listed in Table 4. The remaining 51 compounds were identified at Level 2 (high-resolution MS/MS fragment identification).

### 2.4. Classification of Chemical Constituents in Total Flavonoids from Vine Tea Leaves

**Figure 5:**
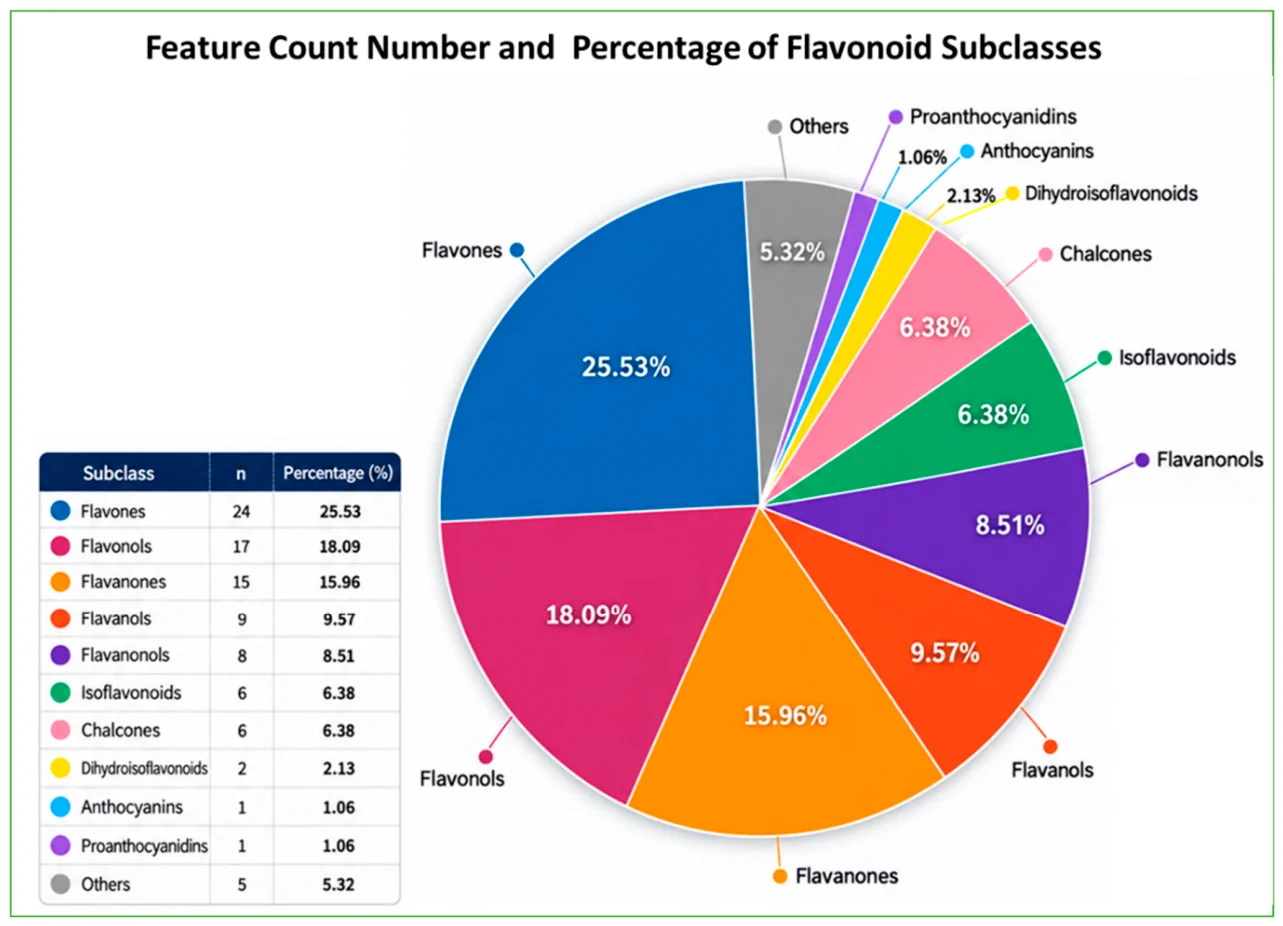
Feature-count distribution of flavonoid subclasses among the 94 compounds identified from vine tea leaves using UPLC-QTOF-MS/MS. The percentage represents the number of features in each subclass divided by the total detected features.

Figure 5 summarizes the distribution of 94 detected flavonoid-related MS features by feature count after the quality review described above. Flavones were the predominant components among the total flavonoids in vine tea leaves, accounting for 25.53% of the identified compounds. Flavonols were the second most abundant class, representing 18.09%, followed by flavanones (15.96%), flavanols (9.57%), and flavanonols (8.51%). Flavanols accounted for 9.47%. Isoflavonoids and chalcones each represented 6.38%, dihydroflavonols represented 2.13%, anthocyanins and proanthocyanidins each represented 1.06%, and other flavonoid subclasses accounted for 5.32%.

### 2.5. Antioxidant Activity of Total Flavonoids from Vine Tea Leaves

**Figure 6:**
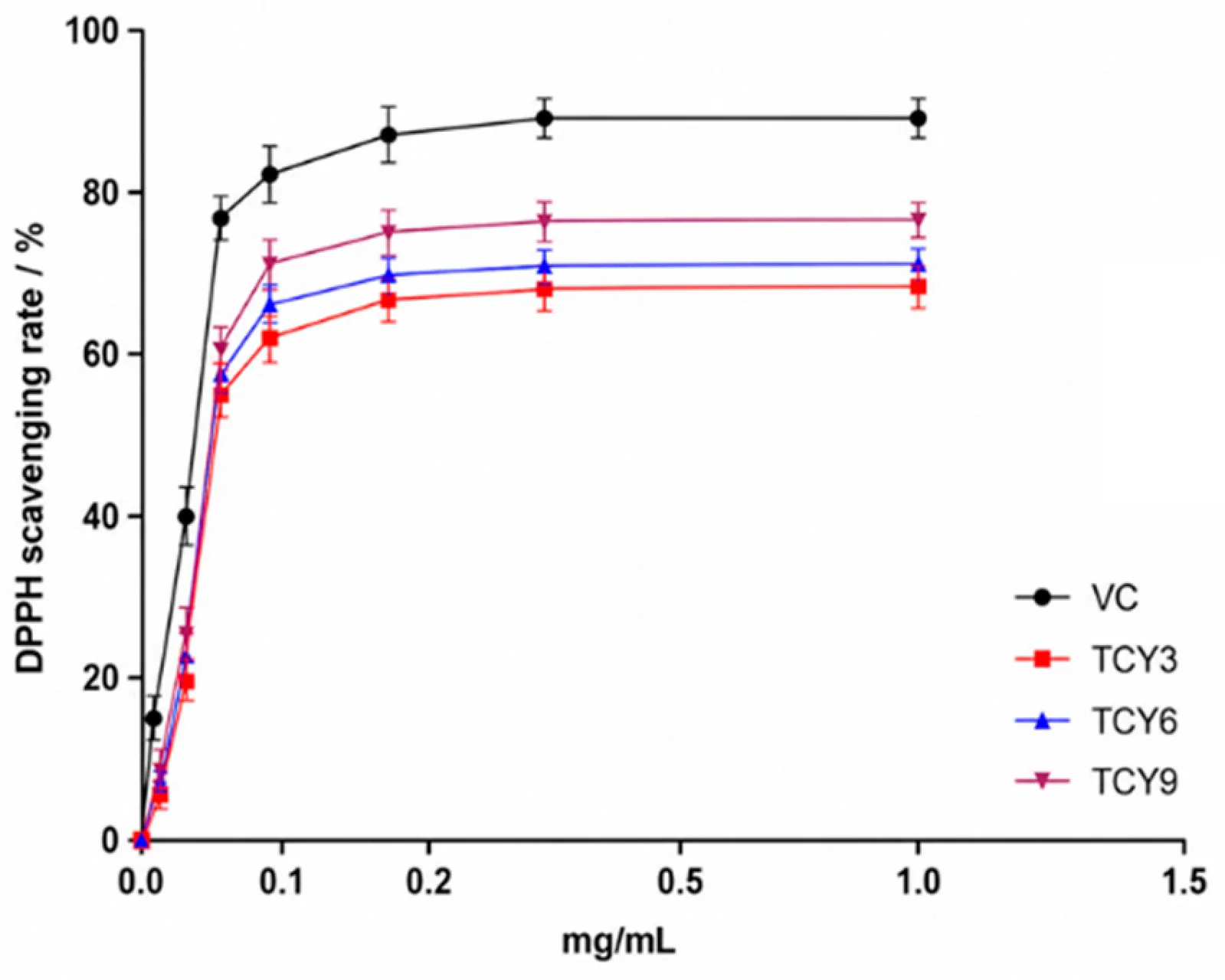
DPPH radical scavenging rates of VC and vine tea leaf flavonoids at different concentrations.

**Figure 7:**
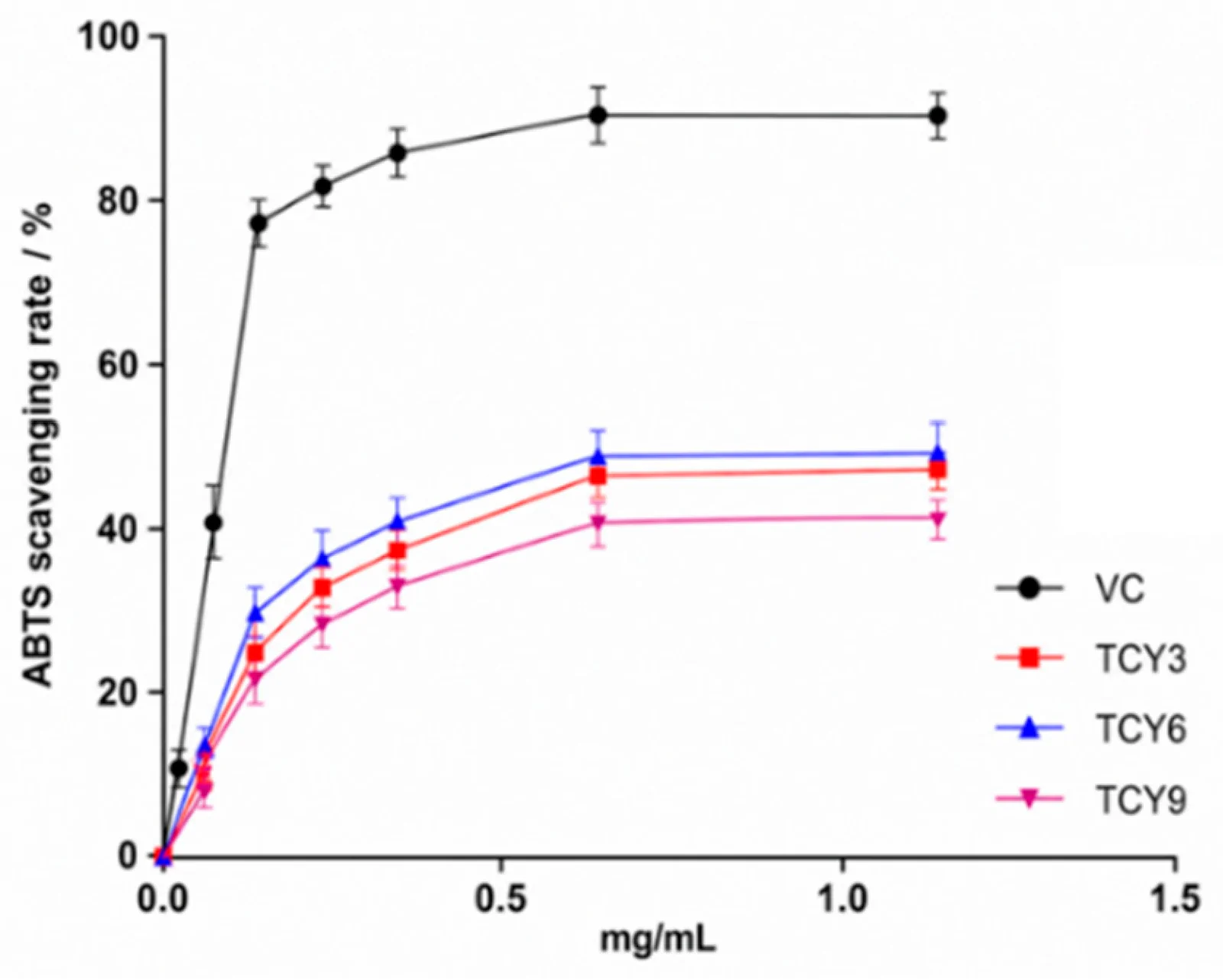
ABTS radical scavenging rates of VC and vine tea leaf flavonoids at different concentrations.

As shown in Figure 6 and Figure 7, the DPPH and ABTS^+^ radical scavenging rates of TCY3, TCY6, and TCY9 increased with increasing sample concentrations. The scavenging activities increased rapidly within the concentration range of 0.005–0.25 mg/mL, while the increase became relatively slow within the range of 0.25–1 mg/mL. The total flavonoids obtained using the three extraction processes exhibited measurable antioxidant activities. Compared with the positive control vitamin C (VC), the scavenging rate curves of TCY3, TCY6, and TCY9 were generally lower over the tested concentration range. However, because IC50 values and 95% confidence intervals were not determined in the present study, the relative antioxidant potency of TCY3, TCY6, TCY9 and VC cannot be quantitatively or statistically compared. Accordingly, Figure 6 and Figure 7 should be interpreted as providing qualitative evidence of concentration-dependent radical scavenging activity rather than a quantitative ranking of antioxidant potency.

### 2.6. Antibacterial Activity of Total Flavonoids from Vine Tea Leaves

As shown in Table 5, TCY3, TCY6, and TCY9 exhibited inhibition zones against *E. coli*, *Salmonella* spp., and *S. aureus* under the tested conditions. *S. aureus* generally showed the greatest susceptibility. Because the observed inhibition zones were small to moderate and were not benchmarked against antibiotic inhibition-zone values in the same table, these results are more appropriately described as indicating antibacterial activity rather than strong antibacterial activity.

### 2.7. Minimum Inhibitory Concentration (MIC) and Minimum Bactericidal Concentration (MBC) of Total Flavonoids from Vine Tea Leaves

As shown in Table 6, Table 7 and Table 8, the MIC and MBC values of TCY3 against *E. coli* were 12.5 mg/mL and 50 mg/mL, respectively; those against *Salmonella* spp. were 6.25 mg/mL and 12.5 mg/mL, respectively; and those against *S. aureus* were both 12.5 mg/mL. For TCY6, the MIC and MBC values against *E. coli* were 6.25 mg/mL and 12.5 mg/mL, respectively; those against *Salmonella* spp. were 6.25 mg/mL and 12.5 mg/mL, respectively; and those against *S. aureus* were 12.5 mg/mL and 50 mg/mL, respectively. For TCY9, the MIC and MBC values against *E. coli* were 6.25 mg/mL and 12.5 mg/mL, respectively; those against *Salmonella* spp. were 12.5 mg/mL and 50 mg/mL, respectively; and those against *S. aureus* were 12.5 mg/mL and 50 mg/mL, respectively.

### 2.8. Cytotoxicity Assessment of Total Flavonoids from Vine Tea Leaves (A. grossedentata)

**Figure 8:**
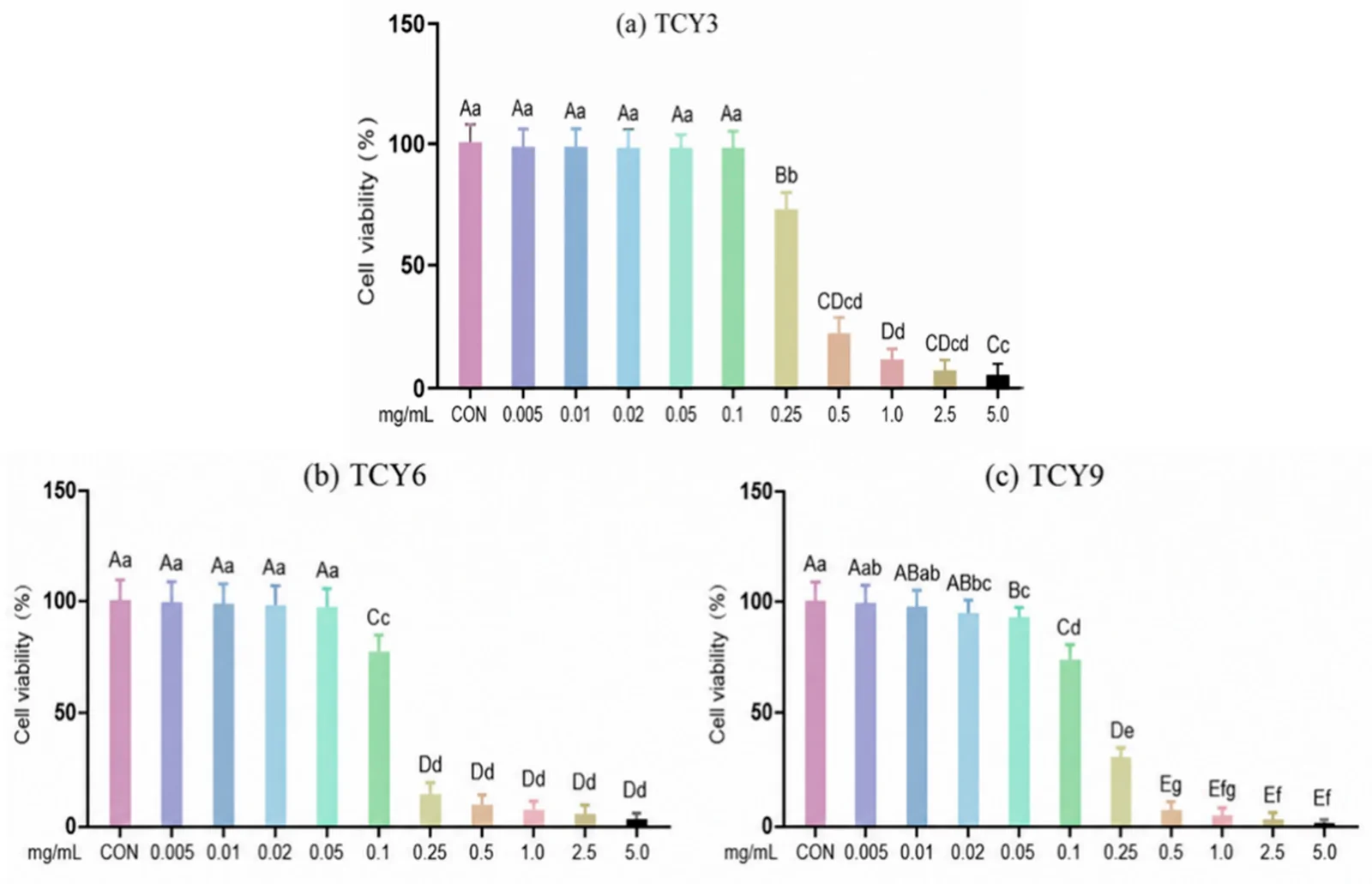
Effect of total flavonoids from vine tea leaves (*A. grossedentata*) on the viability of RAW264.7 cells. Different uppercase letters indicate extremely significant differences (*p* < 0.01), while different lowercase letters indicate significant differences (*p* < 0.05). The same applies below.

As shown in Figure 8, the CCK-8 assay was used to determine the maximum non-cytotoxic concentrations of TCY3, TCY6, and TCY9 across a range of tested concentrations in RAW264.7 cells. The results showed that the maximum non-cytotoxic concentrations of TCY3, TCY6, and TCY9 were all 0.02 mg/mL. Therefore, this concentration was selected as the treatment concentration for subsequent experiments.

### 2.9. Effect of Total Flavonoids from Vine Tea Leaves (A. grossedentata) on NO Production in LPS-Induced RAW264.7 Cells

**Figure 9:**
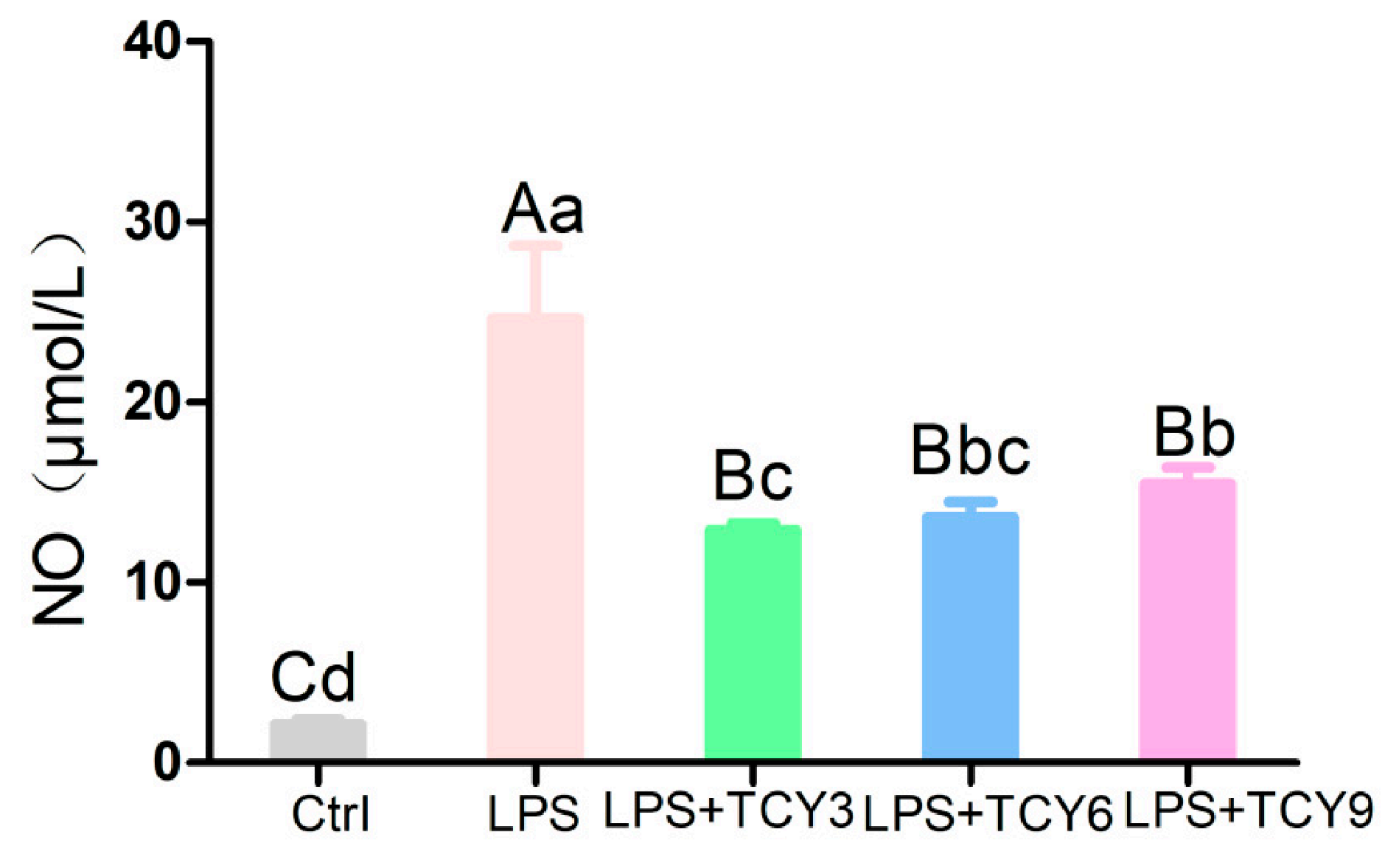
Effect of total flavonoids from vine tea leaves (*A. grossedentata*) on NO production in LPS-induced RAW264.7 cells. Different uppercase letters indicate extremely significant differences (*p* < 0.01).

As shown in Figure 9, the NO content in RAW264.7 cells treated with LPS was significantly increased compared with that in the control group (*p* < 0.01). Compared with the LPS-treated group, the NO content in RAW264.7 cells treated with TCY3, TCY6, and TCY9 was significantly decreased (*p* < 0.01).

### 2.10. Effect of Total Flavonoids from Vine Tea Leaves (A. grossedentata) on Antioxidant Parameters in LPS-Induced RAW264.7 Cells

**Figure 10:**
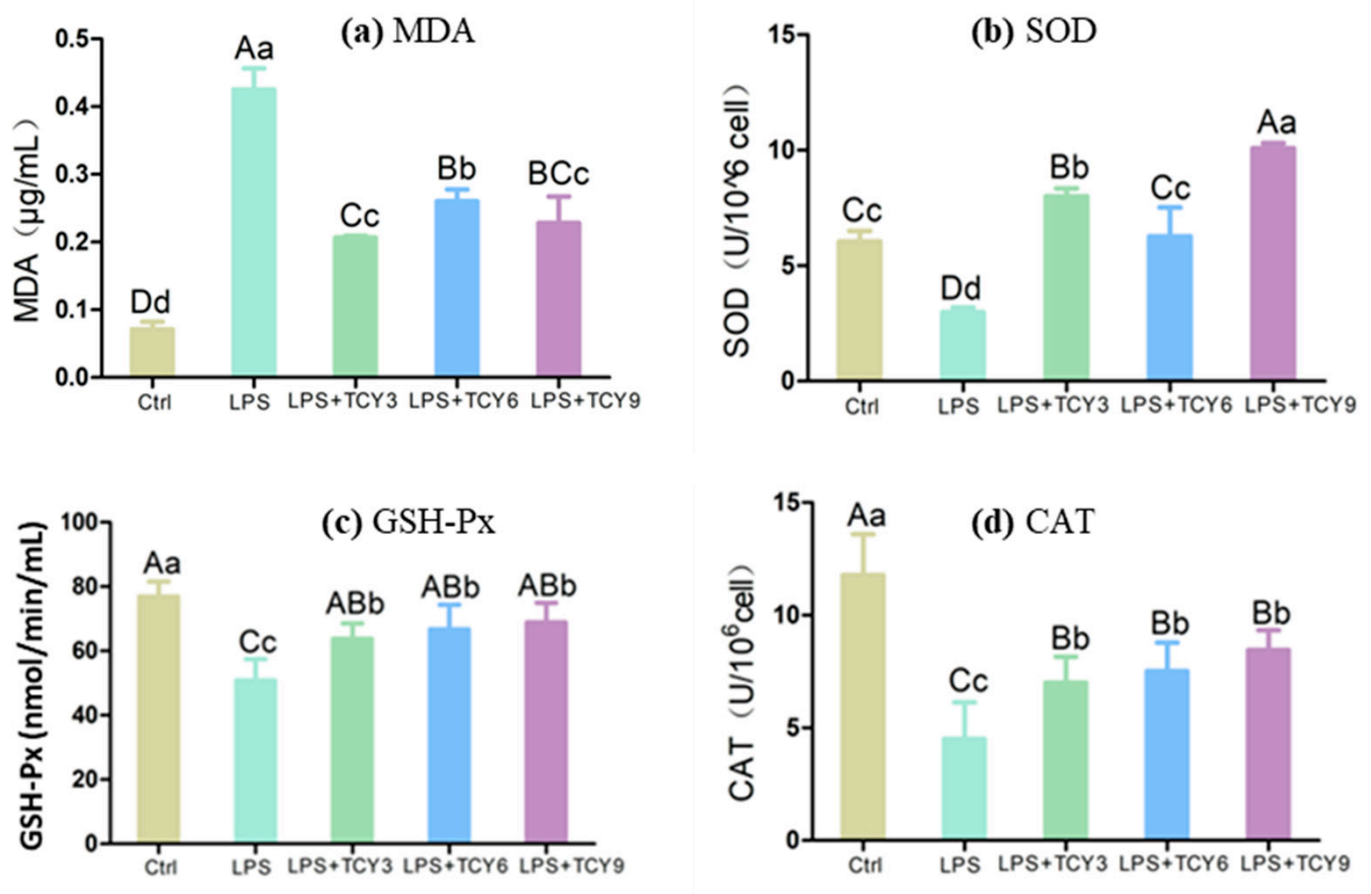
Effect of total flavonoids from vine tea leaves (*A. grossedentata*) on antioxidant parameters in LPS-induced RAW264.7 cells. Different uppercase letters indicate extremely significant differences (*p* < 0.01).

As shown in Figure 10, compared with the control group, the MDA content in the LPS-treated group was significantly increased (*p* < 0.01). Compared with the LPS-treated group, the MDA content in the TCY3, TCY6, and TCY9 groups was significantly decreased (*p* < 0.01). Compared with the control group, the activities of GSH-Px, SOD, and CAT in the LPS-treated group were significantly reduced (*p* < 0.01). Treatment with TCY3, TCY6, and TCY9 significantly increased the activities of GSH-Px, SOD, and CAT compared with the LPS-treated group (*p* < 0.01).

### 2.11. Effect of Total Flavonoids from Vine Tea Leaves (A. grossedentata) on Inflammatory Cytokines in LPS-Induced RAW264.7 Cells

**Figure 11:**
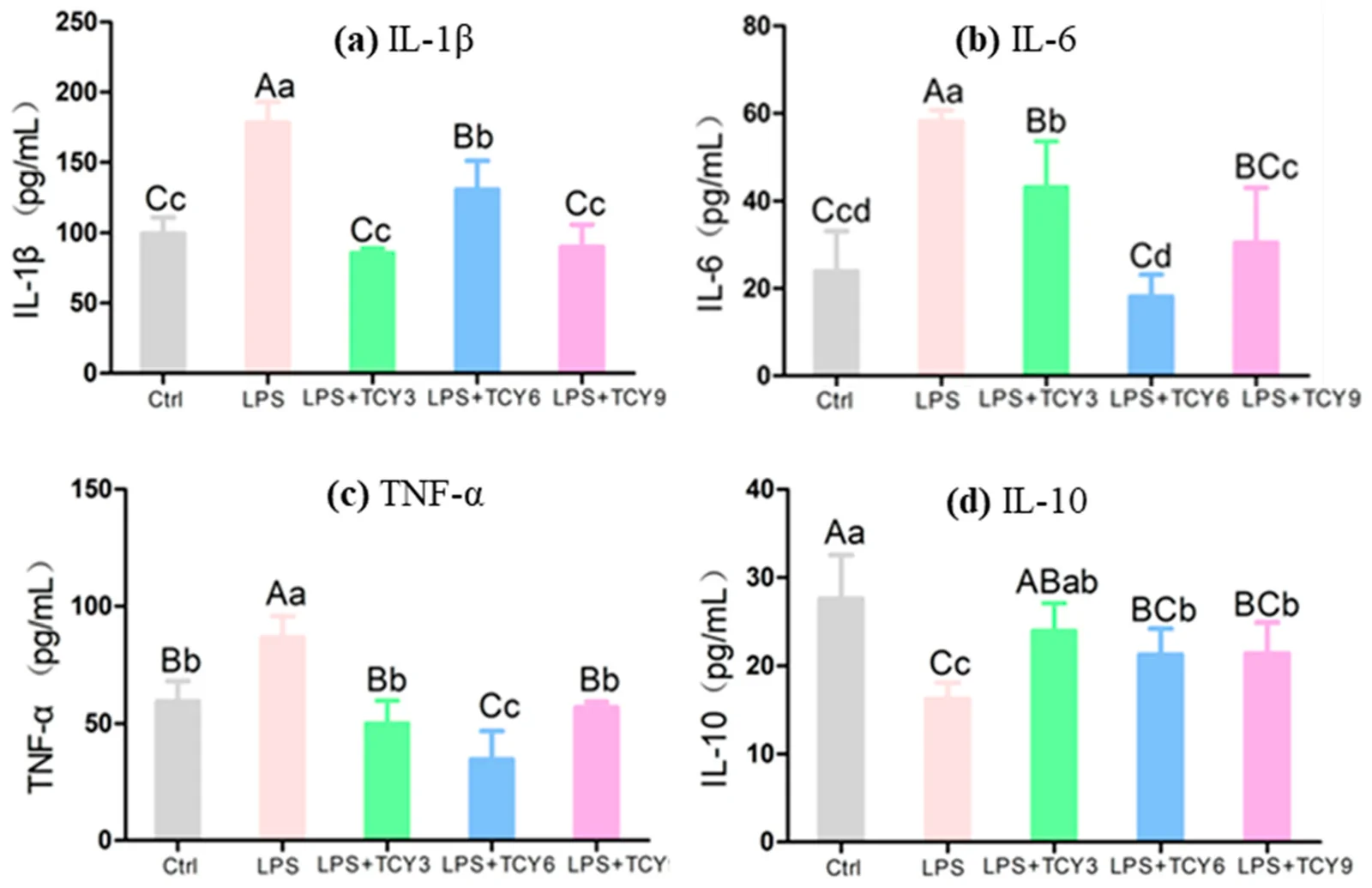
Effect of total flavonoids from vine tea leaves (*A. grossedentata*) on the levels of inflammatory cytokines in LPS-induced RAW264.7 cells. Different uppercase letters indicate extremely significant differences (*p* < 0.01).

As shown in Figure 11, compared with the control group, the levels of IL-1β, IL-6, and TNF-α in the LPS-treated group were significantly increased (*p* < 0.01). Compared with the LPS-treated group, the levels of IL-1β, IL-6, and TNF-α in the TCY3, TCY6, and TCY9 groups were significantly decreased (*p* < 0.01). In contrast, compared with the control group, the IL-10 level in the LPS-treated group was significantly decreased (*p* < 0.01), while treatment with TCY3, TCY6, and TCY9 significantly increased the IL-10 level compared with the LPS-treated group (*p* < 0.01).

## 3. Discussion

Extraction efficiency reflects a balance among solvent polarity, matrix swelling, diffusion, and thermal or acoustic mass transfer. In the present design, the solid-to-liquid ratio had the largest R value for both extraction methods, indicating that solvent availability was the dominant factor within the test range. Increasing the ratio can maintain a favorable concentration gradient and reduce local saturation, but excessive solvent increases downstream evaporation and recovery cost. The ethanol concentration also influenced heat-reflux extraction, consistent with the mixed polarity of flavonoid aglycones and glycosides. The single-factor experiment used a material-to-liquid ratio of 1:10 as a practical central level because the improvement in extraction yield diminished beyond this ratio. The subsequent analysis of level means in the orthogonal experiment also favored a ratio of 1:10. These choices therefore correspond to different stages of the optimization process and should not be interpreted as contradictory optima. For heat reflux extraction, A2B3C2 (70% ethanol, a material-to-liquid ratio of 1:10, and an extraction time of 60 min) was both the theoretically predicted and experimentally observed optimal combination within the L9 array, yielding 58.20 mg RE/g. TCY3 and TCY9 were retained only as additional high-yield extracts for subsequent biological comparisons. The lack of independent replication of the L9 extraction runs represents an important limitation of the present study. Therefore, confirmation experiments using independently replicated batches are required before these conditions can be considered fully validated for scale-up. Similarly, vine tea extracted with 75% ethanol showed the highest content of total flavonoids (15.43%), whereas the n-butanol and ethyl acetate extracts contained 13.90% and 14.96%, respectively [16]. Another study reported that vine tea leaves extracted with 40% ethanol, at an extraction temperature of 50 °C, and with an extraction time of 180 min, yielded the highest total flavonoid content of 40.01 µg CEg^−1^ dry weight [17]. This may be attributed to the differences in solvent polarity at different ethanol concentrations, which affect the solubility of total flavonoids in solvents of varying polarities.

To elucidate the chemical basis of the total flavonoids in vine tea leaves, the extract was subjected to non-targeted chemical profiling using high-resolution mass spectrometry (HRMS). Mass spectral data were acquired in both positive and negative ionization modes, and compounds were identified based on accurate mass measurements, MS/MS fragmentation patterns, and database matching. A total of 94 compounds were identified, including 43 compounds assigned with Level 1 confidence based on accurate mass matching and confirmed identification using authentic reference standards, and 51 compounds assigned with Level 2 confidence based on MS/MS fragment matching. In terms of chemical classification, flavones (24.21%), flavonols (20.00%), and flavanones (16.84%) constituted the three major classes of compounds. Notably, dihydromyricetin, the characteristic marker compound of vine tea leaves, was unequivocally identified [18,19]. The detection of dihydromyricetin is particularly important, because this flavonoid is considered one of the principal phytochemical constituents associated with *A. grossedentata* and is frequently used to characterize the chemical composition and quality of vine tea. In addition to dihydromyricetin, several well-established bioactive compounds, including myricetin, quercetin, luteolin, apigenin, and anthocyanins, were identified [20,21,22,23]. These compounds possess different flavonoid skeletons and substitution patterns, which may result in distinct physicochemical properties and biological activities. These findings provide an important chemical basis for the targeted extraction and utilization of bioactive constituents from vine tea leaves.

The extracts demonstrated pronounced antioxidant activity against both DPPH and ABTS^+^ radicals, as well as substantial ferric reducing antioxidant power (FRAP). These antioxidant effects were strongly associated with the flavonoid content of the extracts, suggesting that flavonoids may contribute significantly to their overall antioxidant capacity. Similarly, *Sonneratia caseolaris* (L.) Engl fruit powder extracts with 70% ethanol yielded the highest total flavonoid content of 19.33 mg/g of dry weight, and these extracts exhibited strong antioxidant activity with DPPH and ABTS values of 151.90 mg TE/g and 184.04 mg AA/g, respectively [24]. Likewise, flavonoid-rich extracts from *Cnidoscolus diacanthus* (Pax & K. Hoffm.) J.F. Macbr showed strong antioxidant activity as determined by DPPH and ABTS^+^ radicals and the FRAP assay [25].

The concentration-dependent DPPH and ABTS responses indicate that all three extracts contain constituents capable of electron or hydrogen donation. Phenolic hydroxyl groups can stabilize radical intermediates by resonance, providing a plausible chemical basis for these assays [26]. In the present study, TCY9 exhibited the lowest antioxidant activity compared with TCY3 and TCY6. This finding may be attributed to its lower flavonoid content. Consistent with previous results, Audah KA et al. [27] reported that a reduction in total flavonoid content leads to decreased antioxidant activity, as flavonoids are among the major contributors to antioxidant capacity. However, total flavonoid content alone cannot explain antioxidant performance because hydroxylation pattern, glycosylation, and interactions among constituents can alter radical-scavenging capacity. The present data therefore support an association, not a causal composition-activity relationship. Quantitative profiling and correlation or multivariate analysis across replicated extracts would be needed to identify the compounds that contribute most strongly to antioxidant activity.

The extracts gave the highest antibacterial activity against *S. aureus* (Gram-positive), with the zone of inhibition exceeding 16.0 mm at the MIC of 12.5 mg/mL. In contrast, the inhibitory effects against *Salmonella* spp. and *E. coli* were comparatively weaker. Similarly, Xiao X-N et al. [28] demonstrated that flavonoids extracted from vine tea leaves significantly inhibited five foodborne pathogens, including three Gram-negative bacteria, *P. aeruginosa, E. coli*, and *S. paratyphi*, and two Gram-positive bacteria, *Bacillus subtilis* and *S. aureus,* at a concentration of 3.78 mg/mL. Similarly, vine tea flavonoid extracts exhibited inhibitory effects against *S. aureus*, *B. cereus*, and *B. pumilus*, as well as the Gram-negative bacteria *E. coli* and *P. fluorescens*. Among these, *S. aureus* was the most sensitive, with a zone of inhibition of 19.30 mm observed at an MIC of 16 mg/mL, compared with the other tested pathogens [29]. The antibacterial activity observed in the extracts is likely associated with the combined action of several bioactive metabolites, particularly flavonoids and other phenolic compounds. These compounds may exert antibacterial effects through multiple mechanisms, including disruption of bacterial cell membranes, alteration of membrane permeability, inhibition of essential microbial enzymes, interference with energy metabolism, chelation of metal ions, inhibition of nucleic acid synthesis, and disturbance of protein function [30]. Differences in antibacterial activity among extracts may therefore reflect variations in the concentration, chemical structure, polarity, and synergistic interactions of their constituent compounds. Differences in susceptibility between bacterial strains may additionally arise from variations in cell-envelope structure, membrane permeability, efflux mechanisms, and other intrinsic resistance characteristics [31].

The greater susceptibility of Gram-positive bacteria may be associated with their simpler cell wall structure, which makes them more susceptible to flavonoid compounds. Previous studies have suggested that the antibacterial activity of flavonoids may function through diverse mechanisms, including impairment of membrane integrity, inhibition of DNA gyrase activity, and interference with cell wall synthesis [32]. This is consistent with the results of Wu Y et al. [33], who observed pronounced morphological abnormalities in bacteria treated with flavonoids from vine tea leaves, including cell wall degradation or loss and leakage of intracellular contents. These observations suggest that the antibacterial activity of treatment with flavonoids from vine tea leaves is partly attributable to their direct disruption of cell wall integrity, resulting in loss of cell viability. Additionally, Liu Wangjing LW et al. [34] demonstrated that flavonoids from vine tea leaves disrupt bacterial membrane integrity, reduce membrane selectivity, and induce the leakage of intracellular components, including nucleic acids, polysaccharides, and metabolites. The pronounced inhibitory activity of total flavonoids from vine tea leaves against S. aureus observed in this study suggests their potential application in controlling infections caused by Gram-positive bacteria.

The LPS-induced RAW264.7 macrophage inflammation model is a well-established in vitro system for evaluating anti-inflammatory activity. Oxidative stress markers were measured using biochemical assay kits: Malondialdehyde (MDA) reflects the extent of lipid peroxidation, whereas catalase (CAT), superoxide dismutase (SOD), and glutathione peroxidase (GSH-Px) represent the activities of endogenous antioxidant enzymes [35,36]. This study showed that total flavonoids from vine tea leaves (TCY3, TCY6, and TCY9) significantly reduced MDA levels in the inflammatory model cells, while markedly increasing the activities of CAT, GSH-Px, and SOD. These findings suggest that total flavonoids from vine tea leaves can effectively alleviate LPS-induced oxidative stress, likely by modulating the balance of the cellular redox system. Literature reports indicated that flavonoid compounds can significantly reduce inflammatory cytokine levels, thereby alleviating oxidative stress, preventing DNA damage, and inhibiting apoptosis-mediated cell death, ultimately contributing to the prevention or amelioration of kidney injury and disease [37,38,39]. However, the present study did not perform comet assays, Annexin V/PI flow cytometry, or other direct measurements of DNA damage or apoptosis, and no such mechanism can be concluded from these data. LPS, a major structural component of the outer membrane of Gram-negative bacteria, interacts with the TLR4 receptor on the cell surface, thereby activating both MyD88-dependent and MyD88-independent signaling pathways. This activation promotes the nuclear translocation of key transcription elements, such as MAPK and NF-κB, leading to the rapid expression of anti-inflammatory cytokines, including IL-6, IL-1β, and TNF-α. Meanwhile, negative feedback regulatory mechanisms are initiated to promote the expression of pro-inflammatory cytokines, including IL-10, thereby maintaining the dynamic balance of the inflammatory response and preventing excessive inflammatory damage [40]. IL-6, IL-1β, and TNF-α are key initiators of the inflammatory cascade. TNF-α activates endothelial cells, recruits immune cells, and induces the production of other pro-inflammatory mediators. IL-1β acts synergistically with TNF-α to amplify inflammatory signaling and contributes to systemic inflammatory responses, including fever and pain. IL-6 is a key mediator of acute inflammation and regulates Th17 cell differentiation. IL-10 is one of the most important endogenous anti-inflammatory cytokines. It exerts its effects by inhibiting NF-κB activity, reducing the transcription of pro-inflammatory cytokines, negatively regulating M1 macrophage polarization, and promoting regulatory T cell differentiation [41].

Nitric oxide (NO) and cytokines are typical indicators of the inflammatory response. It has been reported that LPS stimulation of RAW264.7 cells induces excessive NO production, which can lead to cytotoxicity and trigger inflammatory responses [42]. The results indicated that, compared with the LPS model group, treatment with vine tea leaf flavonoids significantly decreased pro-inflammatory (IL-1β, IL-6, and TNF-α) and NO levels, while increasing IL-10 levels (*p* < 0.01). Notably, TCY6 exhibited stronger inhibitory effects than TCY3 and TCY9 for some inflammatory indicators, suggesting that the anti-inflammatory activity of the extracts may depend not only on their total flavonoid content, but also on differences in flavonoid composition and relative abundance. The observed differences among TCY3, TCY6, and TCY9 may be related to the combined contributions of dihydromyricetin, the major flavonoid detected in the vine tea samples, and other bioactive flavonoids such as myricetin and quercetin. Mechanistically, these compounds share a polyhydroxylated flavonoid scaffold, differing in the number and position of hydroxyl groups, particularly the hydroxyl groups on the B-ring [43,44,45,46]. Dihydromyricetin contains multiple hydroxyl groups that may contribute substantially to the anti-inflammatory activity of the extracts, whereas quercetin and myricetin possess different hydroxylation patterns and degrees of unsaturation that could alter their biological effects. Consequently, variation in the relative proportions of these flavonoids may partly explain why TCY6 exhibited greater activity than TCY3 and TCY9 for certain inflammatory markers. Interactions among these constituents, including additive or synergistic effects, are also possible; however, the present qualitative UPLC-MS metabolomic data do not permit such interactions to be established or the contribution of individual compounds to be quantitatively distinguished. Further studies using quantitative compositional analysis, purified compounds, and defined flavonoid combinations are therefore required to clarify the SAR and determine whether synergistic interactions account for the differences among the three extracts. The macrophage experiments were performed at 0.02 mg/mL, which was the highest concentration considered non-cytotoxic based on the CCK-8 assay. Although this concentration provides an appropriate basis for comparing the extracts under the present in vitro conditions, its physiological relevance remains uncertain. The bioavailability, metabolism, achievable tissue concentrations, and in vivo safety of these flavonoids should therefore be evaluated in subsequent pharmacokinetic and animal studies.

## 4. Materials and Methods

### 4.1. Plant Material

Fresh leaves (*A. grossedentata* (Hand.-Mazz.) W. T. Wang) located 3–5 cm below the tender shoots (the medicinal and edible portion) were collected in Jiangkou village, Tongren City, Guizhou Province, China, on 20 July 2025. The plant material was authenticated by Professor Lihui Zhang (School of Pharmaceutical Sciences, Tongren Polytechnic University). A voucher specimen was deposited at the School of Pharmaceutical Sciences, Tongren Polytechnic University, for future reference.

### 4.2. Reagents

Rutin reference standard (98% purity) was purchased from the National Institutes for Food and Drug Control (China). Lipopolysaccharide (LPS; L2310) was obtained from Sigma-Aldrich. 1,1-Diphenyl-2-picrylhydrazyl (DPPH) and 2,2′-azino-bis (3-ethylbenzothiazoline-6-sulfonic acid) diammonium salt (ABTS) were purchased from Nanjing Jiancheng Bioengineering Institute Co., Ltd, Nanjing, China. A total flavonoid (TF) assay kit (G0118W) was obtained from Grace Biotechnology Co., Ltd., Suzhou, China. Dulbecco’s Modified Eagle Medium (DMEM; 11965092) and fetal bovine serum (FBS; A5670501) were sourced from Gibco (Grand Island, NY, USA). Cell viability was assessed using the Cell Counting Kit-8 (CCK-8; BS350B), which was supplied by Biosharp (Hefei, China). The nitric oxide (NO) detection kit (A013-2-1) was obtained from Nanjing Jiancheng Bioengineering Institute Co., Ltd Nanjing, China. Enzyme-linked immunosorbent assay (ELISA) kits for the quantification of interleukin (IL)-1β (ml106733), IL-10 (ml037873), IL-6 (ml063160), and tumor necrosis factor-α (TNF-α; ml002095) were purchased from Shanghai Enzyme-linked Biotechnology Co., Ltd., Shanghai, China. The same supplier also provided assay kits for evaluating total antioxidant capacity (T-AOC; ml094999), malondialdehyde (MDA; ml094963), superoxide dismutase (SOD; ml092620), catalase (CAT; ml095173), and glutathione peroxidase (GSH-Px; ml095262). Ascorbic acid (A800296, 25 g), penicillin (P990242, 10 mg), and streptomycin (S875203, 250 mg) were purchased from Macklin Biochemical Co., Ltd., Shanghai, China. All remaining reagents were of analytical grade and purchased from commercial suppliers.

### 4.3. Instruments

The instruments used in this study included a U-3900 UV–visible spectrophotometer (Hitachi High-Tech Science Corporation, Tokyo, Japan), JSB30-1 electronic balance (Shanghai Puchun Metrological Instrument Co., Ltd., Shanghai, China), OSB-2200 thermostatic water bath (Grant Instruments, Cambridgeshire, UK), N-1300 rotary evaporator (EYELA, Tokyo, Japan), SB25-12DTD ultrasonic extractor (Ningbo Scientz Biotechnology Co., Ltd., Shanghai, China), F-060SD ultrasonic cleaner (Shenzhen Fuyang Technology Group Co., Ltd., Shenzhen, China), TYXH-1 vortex mixer (Shanghai Hanuo Instrument Co., Ltd., Shanghai, China), TGL-16MS benchtop high-speed refrigerated centrifuge (Shanghai Lu Xiangyi Centrifuge Instrument Co., Ltd., Shanghai, China), ACQUITY UPLC I-Class PLUS system (Waters Corporation, Milford, MA, USA), and Q Exactive Orbitrap high-resolution liquid chromatography–mass spectrometry system (Thermo Fisher Scientific, Waltham, MA, USA).

### 4.4. Experimental Methods

#### 4.4.1. Preparation of Vine Tea Leaves

An appropriate quantity of vine tea leaves was ground into powder, passed through an 80-mesh sieve, sealed, and stored at 4 °C until further analysis.

#### 4.4.2. Determination of Total Flavonoid Content

The total flavonoid content was determined according to the method described by Zhu H et al. [15]. An appropriate amount of rutin was dissolved in 60% ethanol to prepare a 0.50 mg/mL stock solution. Aliquots of 0.4, 0.8, 1.2, 1.6, and 2.0 mL of the rutin stock solution were accurately transferred into separate 10 mL volumetric flasks. Each solution was brought to 5 mL with 60% ethanol, followed by the addition of 0.3 mL of 5% NaNO_2_ solution. After thorough mixing, the solutions were allowed to stand for 6 min. Subsequently, 0.3 mL of 10% aluminum nitrate nonahydrate [Al(NO_3_)_3_·9H_2_O] solution was added. The mixtures were thoroughly mixed and allowed to stand for another 6 min. Thereafter, 4 mL of 4% NaOH solution was added, and the mixtures were incubated for 15 min for color development. The solutions were then diluted to volume with 60% ethanol. A volume of 200 μL from each color-developed solution was placed into a microplate, and the absorbance was measured at 510 nm. A blank control was prepared using 60% ethanol in place of the sample solution. The linear regression equation for the standard curve is presented in Equation (1):

*Y* = 0.0078*x* − 0.0401 (*R*^2^ = 0.999) (1)

The absorbance values of the vine tea leaf extracts obtained using different extraction methods were measured under the same conditions. The total flavonoid content, expressed as rutin equivalents, was calculated from the rutin calibration curve and the corresponding dilution factor using Equation (2):

(2) $$\mathrm{Total}\;\mathrm{flavonoid}\;\mathrm{content}\;(\mathrm{mg}/g)=\frac{\left(P*V*N\right)}{m}$$

where (P) is the mass concentration of total flavonoids in the solution (mg/mL), (V) is the final volume of the extract (mL), (N) is the dilution factor, and (m) is the mass of the vine tea leaf sample (g).

### 4.5. Preparation of Extracts

#### 4.5.1. Single-Factor Experiments

1. To investigate the effect of the solid-to-liquid ratio on total flavonoid extraction from vine tea leaves, the ethanol concentration was fixed at 70% and the heat-reflux or ultrasound-assisted extraction time at 50 min. Solid-to-liquid ratios of 1:6, 1:8, 1:10, 1:12, and 1:14 (g/mL) were evaluated.
2. To investigate the effect of extraction time, the solid-to-liquid ratio and ethanol concentration were fixed at 1:10 (g/mL) and 70%, respectively. Heat-reflux or ultrasound-assisted extraction was performed for 30, 40, 50, 60, or 70 min.
3. To investigate the effect of ethanol concentration, the heat-reflux or ultrasound-assisted extraction time was fixed at 50 min and the solid-to-liquid ratio at 1:10 (g/mL). Ethanol concentrations of 50%, 60%, 70%, 80%, and 90% were evaluated.

#### 4.5.2. Experimental Design for Extraction Optimization

The orthogonal experimental design provides an efficient approach for evaluating the effects of multiple factors on extraction yield while minimizing the number of experimental trials required. This method also facilitates the determination of optimal extraction conditions and enables assessment of the relative influence of each factor on extraction efficiency. Based on the results of the single-factor experiments, three factors (ethanol concentration, solid-to-liquid ratio, and extraction time) were selected as the experimental factors, as shown in Table 9. An L9(3^3^) orthogonal experimental design was used to identify the optimal conditions for heat-reflux and ultrasound-assisted extraction, with the total flavonoid extraction yield from vine tea leaves used as the response variable.

### 4.6. Chemical Characterization of Total Flavonoids from Vine Tea Leaves

#### 4.6.1. Sample Preparation

A 100 mg sample was weighed into a 1.5 mL centrifuge tube, and 1 mL of 70% aqueous methanol was added. The mixture was vortexed for 1 min, and a stainless-steel bead was added. After precooling at −40 °C for 2 min, the sample was homogenized at 60 Hz for 2 min. The homogenate was centrifuged at 12,000 rpm for 10 min at 4 °C, and 300 μL of the resulting supernatant was collected and evaporated to dryness under reduced pressure using a rotary evaporator at 50 °C with a pressure ranging from −0.1 to 0.05 MPa. The dried residue was reconstituted in 150 μL of 70% aqueous methanol and centrifuged for 30 s. A 120-μL aliquot of the supernatant was transferred into an LC–MS autosampler vial fitted with an insert for subsequent analysis.

#### 4.6.2. Chromatographic Conditions

Chromatographic separation was carried out using an ACQUITY UPLC HSS T3 column (100 mm × 2.1 mm, 1.8 μm) kept at 45 °C. The mobile phases consisted of water containing 0.1% formic acid (solvent A) and acetonitrile (solvent B). The flow rate was 0.35 mL/min, and the injection volume was 2 μL. The gradient elution program is presented in Table 10.

#### 4.6.3. Mass Spectrometry Conditions

A heated electrospray ionization (HESI) source was employed. Mass spectrometric data were collected in both positive and negative ionization modes using a data-dependent acquisition (DDA) approach with a Full MS/dd-MS^2^ (Top 10) scan method. The Mass spectrometry conditions is shown in Table 11.

### 4.7. In Vitro Antioxidant Activity Assays of Total Flavonoids from Vine Tea Leaves

#### 4.7.1. Determination of DPPH Radical Scavenging Activity

Five milligrams of each of the TCY3, TCY6, and TCY9 samples was weighed. Each sample was dissolved in absolute ethanol to prepare test solutions with concentrations of 1.0, 0.5, 0.25, 0.1, 0.05, 0.025, and 0.005 mg/mL. Separately, 5 mg of vitamin C (VC) was weighed, and solutions were prepared at the same concentration gradient using the same method for the positive control. These solutions were used for the determination of DPPH radical scavenging activity. A 100-μL aliquot of DPPH solution (0.1 mmol/L) was thoroughly mixed with 50 μL of the sample solution, and the mixture was incubated for 30 min at room temperature. The absorbance was read at 517 nm and recorded as the sample absorbance (A_sample_). Absolute ethanol was used instead of the sample solution as the blank control, and the same procedure was followed. The absorbance measured at 517 nm was recorded as the blank absorbance (A_blank_). Each sample was analyzed in triplicate. The DPPH radical scavenging activity of the total flavonoids from vine tea leaves was calculated using the following Equation (3):

(3) $$\mathrm{DPPH}\;\mathrm{scavenging}\;\mathrm{effect}\;\left(\%\right)=\frac{\left(A_{\mathrm{blank}}-A_{\mathrm{sample}}\right)}{A_{\mathrm{blank}}}*100$$

#### 4.7.2. Determination of ABTS Radical Scavenging Activity

Samples were prepared according to the method described in Section 4.7.1. Assay procedure: 170 μL of ABTS working solution was mixed with 20 μL of sample solution, and the mixture was incubated at room temperature for 60 min. The absorbance was measured at 405 nm and recorded as the sample absorbance (A_sample_). Distilled water was used instead of the sample solution as the blank control, and the same procedure was performed. The absorbance value measured at 405 nm was designated as the blank absorbance. A 10 mM Trolox standard solution was diluted with distilled water to obtain concentrations of 0.1, 0.2, 0.4, 0.8, and 1.0 mM for constructing the standard curve. The absorbance corresponding to the intercept of the standard curve (concentration = 0) was recorded as A_0_. Each sample was measured in triplicate. The ABTS radical scavenging activity of the total flavonoids from vine tea leaves was calculated using the following Equation (4):

(4) $$\mathrm{ABTS}\;\mathrm{scavenging}\;\mathrm{activity}\;\left(\%\right)=\frac{\left(A_{0}-A_{\mathrm{sample}}\right)}{\left(A_{0}-A_{\mathrm{blank}}\right)}*100$$

### 4.8. Antibacterial Activity of Total Flavonoids from Vine Tea Leaves

#### 4.8.1. Preparation of Total Flavonoid Solutions

An appropriate amount of total flavonoids extracted from vine tea leaves using different extraction methods was weighed and dissolved in 2 mL of dimethyl sulfoxide (DMSO). The solution was then diluted with phosphate-buffered saline (PBS) to obtain a stock solution with a final concentration of 100 mg/mL for subsequent antibacterial assays.

#### 4.8.2. Determination of the Antibacterial Activity of Total Flavonoids Against *E. coli*, *Salmonella* spp., and *S. aureus* by the Oxford Cup Method

Overnight cultures of *E. coli*, *Salmonella* spp., and *S. aureus* were diluted with sterile physiological saline to a final concentration of 1 × 10^6^ CFU/mL. Subsequently, 100 μL of the bacterial suspension was evenly spread onto nutrient agar plates using a sterile spreader. Four Oxford cups were placed at the designated positions on each agar plate. The stock solution of total flavonoids from vine tea leaves was diluted with PBS to 50 mg/mL and then serially diluted 2-, 4-, 8-, and 16-fold. Penicillin and streptomycin were each prepared at an initial concentration of 200 μg/mL with distilled water and serially diluted two-fold to a 16-fold dilution. A volume of 200 μL of each prepared solution was added into the corresponding Oxford cup. The plates were incubated at 37 °C for 18–24 h, after which the antibacterial activity was evaluated by measuring the inhibition zones.

#### 4.8.3. Determination of the Minimum Inhibitory Concentration (MIC) and Minimum Bactericidal Concentration (MBC) of Total Flavonoids from Vine Tea Leaves

For the broth microdilution assay, 100 μL of PBS was added to each peripheral well of a 96-well microplate to minimize evaporation. A volume of 100 μL of the prepared bacterial suspension was added to the inner wells. Then, 100 μL of the flavonoid stock solution (100 mg/mL) was added to the wells in the second column and subjected to two-fold serial dilutions to obtain final dilution factors of 2, 4, 8, 16, 32, 64, 128, and 256. A positive control (bacterial suspension without flavonoids) and a negative control (medium without bacterial inoculation) were included. All sample and control wells were prepared in triplicate and incubated statically at 37 °C for 24 h. The MIC was defined as the lowest concentration of total flavonoids at which no visible bacterial precipitation or growth was observed. To determine the MBC, 100 μL aliquots from all wells containing concentrations at or above the MIC were spread onto LB agar plates and incubated at 37 °C for 24 h. The MBC was defined as the lowest flavonoid concentration that resulted in fewer than 5 CFU on the agar plate.

### 4.9. In Vitro Anti-Inflammatory Activity of Total Flavonoids from Vine Tea Leaves

#### 4.9.1. Cytotoxicity Assay of Total Flavonoids from Vine Tea Leaves

RAW264.7 cells were cultured in DMEM supplemented with 10% (*v*/*v*) fetal bovine serum (FBS) and 1% (*v*/*v*) penicillin–streptomycin at 37 °C in a humidified incubator with 5% CO_2_. The cells were diluted to a density of 8 × 10^4^ cells/mL, and 100 μL of the cell suspension was seeded into each well of a 96-well plate. The cells were incubated overnight at 37 °C in a 5% CO_2_ incubator. The experiment included a control group and extract-treated groups with final concentrations of 5, 2.5, 1, 0.5, 0.25, 0.1, 0.02, 0.01, and 0.005 mg/mL. After treatment, the cells were further incubated for 24 h. Six replicate wells were prepared for each concentration. The culture medium was then discarded, and 100 μL of DMEM containing 10% CCK-8 reagent was added to each well. The plates were incubated for an additional 1.5 h, and the absorbance at 450 nm was measured using a microplate reader. Cell viability was calculated according to the following Equation (5):

(5) $$\mathrm{Cell}\;\mathrm{viability}\;\left(\%\right)=\frac{\left({\mathrm{OD}}_{\mathrm{sample}}-{\mathrm{OD}}_{\mathrm{blank}}\right)}{{\mathrm{OD}}_{\mathrm{control}}-{\mathrm{OD}}_{\mathrm{blank}}}*100$$

#### 4.9.2. Protective Effect of Total Flavonoids from Vine Tea Leaves on LPS-Induced Inflammation in RAW264.7 Cells

RAW264.7 cells were seeded into 6-well plates at a density of 2 × 10^5^ cells per well and cultured for 16 h to allow cell attachment. The culture medium was then removed, and the cells were divided into the following groups: a control group (without any treatment), an LPS group (RAW264.7 cells stimulated with LPS), and TCY3, TCY6, and TCY9 treatment groups (RAW264.7 cells treated with the maximum non-cytotoxic concentration of TCY3, TCY6, or TCY9 in the presence of LPS). Each group consisted of six replicates. After pretreatment for 4 h, lipopolysaccharide (LPS) was added to all groups except the control group to a final concentration of 1 μg/mL, and the cells were further incubated for 24 h to establish the inflammatory model. After incubation, the cell culture supernatants were collected, and the nitric oxide (NO) levels were determined using a Griess reagent assay kit according to the manufacturer’s instructions. The absorbance was measured at 540 nm using a microplate reader.

#### 4.9.3. Antioxidant Effects of Total Flavonoids from Vine Tea Leaves on LPS-Induced Inflammatory RAW264.7 Cells

RAW264.7 cells were treated according to the procedure described in Section 4.9.2, with six replicates for each group. After treatment, the cells were washed three times with pre-cooled phosphate-buffered saline (PBS). Cell lysis buffer was then added on ice, and the cells were gently scraped using a cell scraper. The collected cells were transferred into 1.5 mL EP tubes, lysed by ultrasonication, and centrifuged at 10,000 rpm for 5 min at 4 °C. The supernatants were collected for subsequent analysis. The levels of malondialdehyde (MDA) and the activities of superoxide dismutase (SOD), catalase (CAT), and glutathione peroxidase (GSH-Px) were determined according to the manufacturer’s instructions using the corresponding assay kits.

#### 4.9.4. Effects of Total Flavonoids from Vine Tea Leaves on Pro-Inflammatory Cytokines in LPS-Induced Inflammatory RAW264.7 Cells

RAW264.7 cells were grouped and treated according to the procedure described in Section 4.9.2, with six replicates for each group. The cell culture supernatants were collected and centrifuged at 1000 rpm for 10 min. The secretion levels of TNF-α, IL-6, IL-10, and IL-1β in the cell culture supernatants were determined according to the instructions of the ELISA kits.

### 4.10. Statistical Analysis

The experiments were conducted in triplicate to ensure the reliability of the results. The experimental data were collected and organized using Microsoft Excel. One-way analysis of variance (ANOVA) was performed using SPSS version 20.0. The results are expressed as the mean ± standard deviation. Differences were considered statistically significant at *p* < 0.05 and highly significant at *p* < 0.01.

## 5. Conclusions

In conclusion, the optimized extraction process established in this study produced vine tea leaf extracts with a total flavonoid content exceeding 50 mg RE/g. The obtained extract was rich in flavonoids, flavonols, dihydroflavonoids, and other compounds, with a total of 94 compounds identified. It exhibited strong in vitro antioxidant activity, significant antibacterial activity against Gram-positive bacteria, and the ability to regulate the oxidative stress–inflammation network in macrophages. The findings support the further development and valorization of vine tea leaf by-products; however, several limitations should be addressed before their industrial or health-related applications can be considered. First, the optimized extraction conditions require independent validation through replicated experiments. In addition, the metabolomics analysis conducted in this study was primarily qualitative and should be complemented by targeted quantitative analyses across independent batches, seasons, and geographic origins to establish the robustness and reproducibility of the chemical profile. Future studies should also evaluate extract stability during storage and processing, in vivo toxicity and maximum tolerated exposure, pharmacokinetics, and the physiological relevance of the concentrations tested. Further investigation of the underlying molecular mechanisms and potential synergistic interactions among the major flavonoids is also warranted. Finally, pilot-scale extraction studies should assess solvent recovery, energy consumption, batch-to-batch reproducibility, product standardization, regulatory requirements, and overall techno-economic feasibility.

## Figures and Tables

**Table 1:** Factors and levels used in the orthogonal experimental design.

Level	Experiment		
	A: Ethanol Concentration (%)	B: Solid-to-Liquid Ratio	C: Heat Reflux/Ultrasonic Extraction Time (min)
1	60	1:08	50
2	70	1:10	60
3	80	1:12	70

**Table 2:** Effects of different ultrasonic extraction conditions on the yield of total flavonoids from vine tea leaves.

Experiment	A: Ethanol Concentration (%)	B: Solid-to-Liquid Ratio	C: Ultrasonic Extraction Time (min)	Total Flavonoid Content (mg RE/g)
1	1	1	1	25.33
2	1	2	2	38.94
3	1	3	3	43.28
4	2	1	3	35.13
5	2	2	1	36.23
6	2	3	2	48.37
7	3	1	2	29.07
8	3	2	3	43.49
9	3	3	1	39.67

**Table 3:** Effects of different heat reflux extraction conditions on the yield of total flavonoids from vine tea leaves.

Experiment	A: Ethanol Concentration (%)	B: Solid-to-Liquid Ratio	C: Heat Reflux/Ultrasonic Extraction Time (min)	Total Flavonoid Content (mg RE/g)
1	1	1	1	33.89
2	1	2	2	38.85
3	1	3	3	53.13
4	2	1	3	41.42
5	2	2	1	46.06
6	2	3	2	58.20
7	3	1	2	46.50
8	3	2	3	42.31
9	3	3	1	51.07

**Table 4:** Chemical constituents identified in vine tea leaves.

No.	Compound Name	Class	Subclass	Adduct	Formula	Theoretical (*m*/*z*)	Fragment Ions	Mass Error (ppm)	Retention Time (min)
1	5,7-Dihydroxychromone	Flavonoids	Isoflavonoids	M−H	C_9_H_6_O_4_	177.01933	177.0191	−3.95	16.871
2	Naringenin	Flavonoids	Flavanones	M−H	C_15_H_12_O_5_	271.0612	119.0485, 151.0036, 271.0599	1.73	21.961
3	Phloretin	Flavonoids	Chalcones	M−H	C_15_H_14_O_5_	273.07685	107.0493, 122.0366, 167.0337, 229.0866, 273.0787	0.4	22.022
4	Eriodictyol	Flavonoids	Flavanones	M−H	C_15_H_12_O_6_	287.05611	107.0124, 125.0231, 135.0439, 151.0035, 287.0218, 287.0584	1.29	20.955
5	Aromadendrin	Flavonoids	Flavanones	M−H	C_15_H_12_O_6_	287.05611	125.0226, 151.0031, 178.9978, 201.0554, 227.1296, 243.0642, 259.0632, 287.0219, 287.0535, 287.1476	1.39	19.756
6	L-Epicatechin	Flavonoids	Flavanols	M−H	C_15_H_14_O_6_	289.07176	109.0279, 125.0233, 179.0345, 203.0701, 245.0830, 289.0374, 289.0691	1.9	13.77
7	Catechin	Flavonoids	Flavanols	M−H	C_15_H_14_O_6_	289.07176	109.0288, 125.0229, 179.0338, 203.0695, 205.0505, 245.0823, 289.0739	1.97	11.423
8	Morin	Flavonoids	Flavonols	M−H	C_15_H_10_O_7_	301.03538	151.0036, 178.9981, 301.0375	0.43	21.177
9	Tricin	Flavonoids	Flavones	M−H	C_15_H_10_O_7_	301.03538	301.0373	0.3	20.408
10	Taxifolin	Flavonoids	Flavanones	M−H	C_15_H_12_O_7_	303.05103	125.0229, 175.0379, 177.0189, 178.9977, 217.0506, 241.0502, 259.0625, 275.0562, 285.0425, 303.0494	−1.65	17.097
11	Epigallocatechin	Flavonoids	Flavanols	M−H	C_15_H_14_O_7_	305.06668	125.0231, 137.0234, 139.0380, 165.0193, 167.0337, 179.0341, 219.0665, 261.0761, 305.0652	1.31	10.936
12	L-Epigallocatechin	Flavonoids	Flavanols	M−H	C_15_H_14_O_7_	305.06668	125.0230, 137.0233, 139.0396, 165.0191, 167.0335, 179.0339, 219.0664, 261.0759, 305.0648	0.2	8.149
13	Myricetin	Flavonoids	Flavonols	M−H	C_15_H_10_O_8_	317.03029	137.0232, 151.0033, 178.9977, 317.0300	−0.63	20.171
14	Irisone A	Flavonoids	Isoflavonoids	M−H	C_17_H_14_O_7_	329.06668	299.0175, 314.0457, 328.2229, 329.0283, 329.0668	−1.61	22.481
15	Luteolin	Flavonoids	Flavones	M+FA−H	C_15_H_10_O_6_	331.04594	108.0202, 121.0282, 123.0444, 152.0106, 167.0337, 305.2767, 329.0889	−0.6	8.574
16	Anthocyanin	Flavonoids	Anthocyanins	M+	C_15_H_11_O_6+_	287.05501	151.0017, 178.9982, 316.0213, 331.0436	0.59	21.224
17	3,3′,4′,7-Tetrahydroxyflavone	Flavonoids	Flavonols	M+H	C_15_H_10_O_6_	287.05501	287.0574, 288.0576	0.66	14.61
18	Kaempferol	Flavonoids	Flavones	M+H	C_15_H_10_O_6_	287.05501	157.0650, 173.0604, 185.0607, 213.0554, 241.0512, 243.0653, 269.0451, 287.0572, 288.0576, 305.0672	0.31	19.781
19	Dihydromyricetin	Flavonoids	Flavanonols	M+H	C_15_H_12_O_8_	321.06049	287.0574, 288.0567	−1.59	22.295
20	Afzelin	Flavonoids	Flavonols	M−H	C_21_H_20_O_10_	431.09837	139.0384, 149.0232, 153.0187, 167.0346, 195.0301, 219.0293, 247.0612, 275.0536, 303.0511, 321.0615	1.86	13.025
21	Persicarin	Flavonoids	Flavonols	M−H	C_20_H_18_O_11_	433.07764	255.0276, 284.0328, 285.0378, 431.1001, 432.1017	2.26	20.497
22	Guaiaverin	Flavonoids	Flavonols	M−H	C_20_H_18_O_11_	433.07764	271.0259, 300.0251, 301.0375, 433.0772	0.99	19.314
23	Naringenin-7-O-glucoside	Flavonoids	Flavanones	M−H	C_21_H_22_O_10_	433.11402	271.0262, 300.0250, 301.0319, 433.0769, 434.0851	2.36	18.918
24	Trilobatin	Flavonoids	Chalcones	M−H	C_21_H_24_O_10_	435.12967	151.0033, 271.0596, 433.1206	1.01	19.597
25	Epicatechin gallate	Flavonoids	Flavanols	M−H	C_22_H_18_O_10_	441.08272	167.0337, 229.0864, 273.0788, 297.0783, 435.1267, 436.1328	−1.52	20.705
26	Quercitrin	Flavonoids	Flavones	M−H	C_21_H_20_O_11_	447.09329	125.0231, 169.0144, 245.0827, 271.0602, 289.0743, 441.0834, 442.0894	−0.16	17.357
27	Neoastilbin	Flavonoids	Flavanonols	M−H	C_21_H_22_O_11_	449.10894	271.0258, 300.0247, 301.0371, 447.0910, 448.0973	1.74	19.753
28	Gallocatechin gallate	Flavonoids	Flavanols	M−H	C_22_H_18_O_11_	457.07764	135.0436, 151.0031, 287.0573, 449.1085, 450.1143	0.44	19.249
29	Myricitrin	Flavonoids	Flavonols	M−H	C_21_H_20_O_12_	463.0882	125.0230, 169.0142, 305.0652, 457.0875	0.52	14.799
30	Taxifolin 7-O-glucoside	Flavonoids	Flavanonols	M−H	C_21_H_22_O_12_	465.10385	271.0260, 316.0207, 317.0304, 463.0886	−0.43	17.413
31	Plantainoside	Flavonoids	Flavanones	M−H	C_21_H_22_O_12_	465.10385	125.0230, 153.0175, 167.0339, 177.0192, 178.9977, 275.0568, 285.0431, 303.0500, 329.0870, 465.1111	−0.56	11.603
32	Epigallocatechin 3-O-(3-O-methyl) gallate	Flavonoids	Flavanols	M−H	C_23_H_20_O_11_	471.09329	151.0018, 151.0395, 303.0496, 313.0919, 465.1087	−1.78	16.667
33	Myricetin-3′-O-β-D-glucoside	Flavonoids	Flavonols	M−H	C_21_H_20_O_13_	479.08311	125.0232, 137.0236, 161.0238, 168.0046, 183.0299, 269.0446, 287.0585, 305.0653, 470.9466, 471.0884	0.58	16.363
34	Phlorizin	Flavonoids	Chalcones	M+FA−H	C_21_H_24_O_10_	481.13515	178.9980, 316.0207, 317.0306, 479.0817, 480.0851	2.37	18.336
35	Rotenone	Flavonoids	Dihydroisoflavonoids	M+H	C_23_H_22_O_6_	395.14891	167.0337, 273.0787, 435.1265	−7.47	20.286
36	Kaempferol-3-O-α-L-arabinopyranoside	Flavonoids	Flavones	M+H	C_20_H_18_O_10_	419.09727	395.1468, 396.1522	−1.93	30.615
37	Epitheaflagallin 3-O-gallate	Flavonoids	Flavanols	M−H	C_27_H_20_O_13_	551.08311	73.0289, 287.0527, 288.0575, 419.0566, 419.1665	2.58	19.985
38	Procyanidin B3	Flavonoids	—	M−H	C_30_H_26_O_12_	577.13515	125.0233, 151.0017, 178.9981, 193.0131, 335.0580, 407.0434, 425.0521, 533.0754, 551.0834	2.98	20.725
39	Procyanidin B4	Flavonoids	Proanthocyanidins	M−H	C_30_H_26_O_12_	577.13515	125.0230, 161.0230, 245.0826, 287.0585, 289.0740, 299.0566, 407.0785, 425.0901, 451.1061, 577.1416	2.72	11.185
40	Isoneohesperidin	Flavonoids	Flavanones	M+Na	C_21_H_22_O_10_	457.11051	125.0229, 208.0242, 208.0467, 264.9782, 289.0678, 305.2940, 407.0792, 425.0904, 451.1077, 577.1350	1.77	12.527
41	Epigallocatechin gallate	Flavonoids	Flavanols	M+H	C_22_H_18_O_11_	459.09219	457.1076, 457.2394, 458.1166	−1.72	16.563
42	Myricetin-3-O-β-D-glucoside	Flavonoids	Flavonols	M+H	C_21_H_20_O_13_	481.09767	319.0451, 481.1016, 481.1695	0	20.224
43	Apigenin	Flavonoids	Flavones	M−H	C_15_H_10_O_5_	269.04555	319.0449	2.12	16.021
44	Pinobanksin	Flavonoids	Flavanonols	M−H	C_15_H_12_O_5_	271.0612		1.66	22.097
45	Eupatilin	Flavonoids	Flavanones	M−H	C_15_H_12_O_6_	287.05611	225.0562, 227.0707, 253.0481, 271.0263, 271.0599	1.36	22.482
46	Quercetin	Flavonoids	Flavonols	M−H	C_15_H_10_O_7_	301.03538		0.2	21.586
47	Dihydromorin	Flavonoids	Flavanonols	M−H	C_15_H_12_O_7_	303.05103	175.0037, 191.0338, 215.0333, 257.0479, 300.9984, 301.0320, 301.0713	−1.25	21.579
48	4,4′-Dihydroxy-2′-methoxychalcone	Flavonoids	Chalcones	M+FA−H	C_16_H_14_O_4_	315.08741		0.76	17.659
49	Dihydrotamarixetin	Flavonoids	Flavanonols	M−H	C_16_H_14_O_7_	317.06668		−2.9	21.52
50	Dihydrodaidzein	Flavonoids	Dihydroisoflavonoids	M+H	C_15_H_12_O_4_	257.08083		−0.58	20.782
51	7,8,3′,4′-Tetrahydroxydihydroflavone	Flavonoids	—	M+FA−H	C_15_H_12_O_6_	333.06159		−0.66	20.703
52	Butein	Flavonoids	Flavanones	M+H	C_15_H_12_O_5_	273.07575		−0.29	16.987
53	Balsaminone A	Flavonoids	—	M−H	C_17_H_14_O_8_	345.06159	147.0446, 153.0188, 171.0297, 273.0755	−0.75	19.586
54	Scutellarein	Flavonoids	Flavones	M+H	C_15_H_10_O_6_	287.05501		1.36	20.871
55	Licochalcone B	Flavonoids	Chalcones	M+H	C_16_H_14_O_5_	287.0914		−6.9	20.484
56	5,7-Dimethoxyluteolin	Flavonoids	Flavones	M+FA−H	C_17_H_14_O_6_	359.07724		0.7	20.877
57	Robinetin	Flavonoids	Flavonols	M+H	C_15_H_10_O_7_	303.04993		−2.24	21.553
58	6-Hydroxyluteolin	Flavonoids	Flavones	M+H	C_15_H_10_O_7_	303.04993		−2.05	17.643
59	Norwogonin-7-O-glucuronide	Flavonoids	Flavones	M−H	C_21_H_18_O_11_	445.07764		−0.13	19.71
60	Apigenin-7-O-glucuronide	Flavonoids	Flavones	M−H	C_21_H_18_O_11_	445.07764		0.49	20.996
61	Astilbin isomer	Flavonoids	—	M−H	C_21_H_22_O_11_	449.10894		2.29	19.819
62	Eriodictyol-7-O-glucoside	Flavonoids	Flavanones	M−H	C_21_H_22_O_11_	449.10894	135.0439, 151.0017, 287.0584, 449.1085, 450.1124	0.38	19.999
63	Oroxin A glucoside	Flavonoids	Flavones	M−H	C_22_H_20_O_11_	459.09329		1.05	17.377
64	Luteolin-3′-glucuronide	Flavonoids	Flavones	M−H	C_21_H_18_O_12_	461.07255	151.0018, 177.0190, 178.9980, 357.0285, 415.2350, 461.0737	0.56	20.37
65	Scutellarin	Flavonoids	Flavones	M−H	C_21_H_18_O_12_	461.07255		0.41	17.511
66	Isorhoifolin	Flavonoids	Flavones	M−H	C_22_H_22_O_11_	461.10894		1.91	18.562
67	6-Hydroxyluteolin glucoside	Flavonoids	Flavones	M−H	C_21_H_20_O_12_	463.0882		0.78	16.673
68	Pinobanksin-7-O-β-D-glucoside (Pinobanksin glucoside)	Flavonoids	Flavanones	M+FA−H	C_21_H_22_O_9_	463.12458		2.76	21.412
69	Liquiritin	Flavonoids	Flavanones	M+FA−H	C_21_H_22_O_9_	463.12458		5.77	17.438
70	Isorhamnetin-3-O-glucoside	Flavonoids	Flavonols	M−H	C_22_H_22_O_12_	477.10385	125.0229, 208.0983, 305.2768, 315.0155, 316.0199, 330.0352, 331.0491, 477.1064, 478.1037	0.1	19.924
71	Isosakuranetin glycoside	Flavonoids	Flavanonols	M+FA−H	C_21_H_22_O_10_	479.1195		2.11	20.577
72	Silybin	Flavonoids	Flavanonols	M−H	C_25_H_22_O_10_	481.11402		−0.08	21.592
73	Juglanin	Flavonoids	Flavones	M+H−H2O	C_20_H_18_O_10_	401.0873		−0.1	20.28
74	6″-O-Malonylglycitin	Flavonoids	Isoflavonoids	M−H	C_24_H_22_O_12_	501.10385		−2.35	19.597
75	Sophoranol A	Flavonoids	Flavanones	M+H	C_25_H_28_O_5_	409.20095		−7.31	32.207
76	Ononin	Flavonoids	Isoflavonoids	M+H−H2O	C_22_H_22_O_9_	413.12368		−4.5	20.607
77	Diosmetin-7-O-β-D-glucuronide	Flavonoids	Flavones	M+FA−H	C_22_H_20_O_12_	521.09368		−1.46	20.497
78	Neokurarinol	Flavonoids	Isoflavonoids	M+H	C_21_H_20_O_9_	417.11801	417.1187, 418.1202	−5.56	17.371
79	Silydianin	Flavonoids	Flavanones	M+FA−H	C_25_H_22_O_10_	527.1195	125.0233, 151.0017, 152.0109, 178.9980, 317.0308, 318.0327, 527.1142	0.42	21.145
80	Isoliquiritin apioside	Flavonoids	Chalcones	M−H	C_26_H_30_O_13_	549.16137		3.35	20.536
81	5-Methoxy-pinobanksin glucoside	Flavonoids	Flavanones	M+H	C_22_H_24_O_9_	433.14931		−3.88	20.142
82	Quercetin-3-O-β-D-xyloside	Flavonoids	Flavones	M+H	C_20_H_18_O_11_	435.09219		0.94	18.554
83	Tiliroside	Flavonoids	Flavonols	M−H	C_30_H_26_O_13_	593.13006		0.98	21.145
84	Trifolin	Flavonoids	Flavonols	M+Na	C_21_H_20_O_11_	471.08978		−1.57	18.955
85	6″-O-Acetylglycitin	Flavonoids	Isoflavonoids	M+H−H2O	C_24_H_24_O_11_	471.12916		−0.72	20.527
86	Vicenin-2	Flavonoids	Flavones	M+FA−H	C_27_H_30_O_15_	639.15667		0.76	14.669
87	Wogonoside	Flavonoids	Flavones	M+Na	C_22_H_20_O_11_	483.08978		5.42	21.43
88	Quercetin-3′-O-glucoside	Flavonoids	—	M+Na	C_21_H_20_O_12_	487.08469		6.61	20.703
89	Pectolinarin	Flavonoids	Flavones	M+Na	C_23_H_24_O_11_	499.12108		2.5	20.924
90	Icaritin-3-O-rutinoside (Icariside V)	Flavonoids	Flavonols	M−H	C_38_H_48_O_19_	807.2717		2.27	20.342
91	6,4′-Methylated scutellarein-7-O-glucuronide	Flavonoids	Flavones	M+Na	C_24_H_24_O_12_	527.11599	160.0518, 192.0787, 233.0419, 289.0322, 305.1871, 317.0282, 331.0822, 355.0421, 509.1032, 527.1205	5.03	22.86
92	Oroxin B	Flavonoids	Flavones	M+H	C_27_H_30_O_15_	595.16575		0.34	19.71
93	Rutin	Flavonoids	Flavonols	M+Na	C_27_H_30_O_16_	633.1426	305.1986, 315.1049, 487.0834, 633.1525	2.21	17.434
94	Quercetin-3-O-(6″-galloyl)-β-D-glucoside	Flavonoids	Flavonols	M+Na	C_28_H_24_O_16_	639.09565	153.0188, 603.0739	1.56	17.226

**Table 5:** Antibacterial activity of total flavonoids from vine tea leaves (unit: mm).

Bacterial Strain	*E. coli*				*Salmonella* spp.				*S. aureus*			
Drug Conc (mg/mL)	50	25	12.5	6.25	50	25	12.5	6.25	50	25	12.5	6.25
TCY3	14.3	0	0	0	10.9	15.4	11.2	11.0	18.4	18.0	17.1	16.2
TCY6	13.4	12.0	0	0	12.9	12.2	14.3	13.9	17.5	17.9	17.3	16.3
TCY9	16.9	12.1	11.7	12.1	12.8	12.2	11.0	12.4	17.2	16.9	16.2	16.2

Note: TCY3, TCY6, and TCY9 represent total flavonoid extracts obtained using three different extraction processes. “0” indicates no detectable inhibition zone. Based on a 100 mg/mL working solution followed by 2-, 4-, 8-, and 16-fold dilution, the corresponding extract concentrations were 50, 25, 12.5, and 6.25 mg/mL, respectively.

**Table 6:** MIC and MBC of TCY3 against different bacterial strains.

Bacteria/Concentration (mg/mL)	50	25	12.5	6.25	3.125	1.56	0.781	Negative Control	Positive Control
*E. coli* (MIC)	−	−	−	+	+	+	+	−	+
*E. coli* (MBC)	−	−	+	+	+	+	+	−	+
*Salmonella* spp. (MIC)	−	−	−	−	+	+	+	−	+
*Salmonella* spp. (MBC)	−	−	−	+	+	+	+	−	+
*S. aureus* (MIC)	−	−	−	+	+	+	+	−	+
*S. aureus* (MBC)	−	−	−	+	+	+	+	−	+

Note: “+” indicates bacterial growth, while “−” indicates inhibition of bacterial growth for MIC and MBC.

**Table 7:** MIC and MBC of TCY6 against different bacterial strains.

Bacteria/Concentration (mg/mL)	50	25	12.5	6.25	3.125	1.56	0.781	Negative Control	Positive Control
*E. coli* (MIC)	−	−	−	−	+	+	+	−	+
*E. coli* (MBC)	−	−	−	+	+	+	+	−	+
*Salmonella* spp. (MIC)	−	−	−	−	+	+	+	−	+
*Salmonella* spp. (MBC)	−	−	−	+	+	+	+	−	+
*S. aureus* (MIC)	−	−	−	+	+	+	+	−	+
*S. aureus* (MBC)	−	−	+	+	+	+	+	−	+

Note: “+” indicates bacterial growth, while “−” indicates inhibition of bacterial growth for MIC and MBC.

**Table 8:** MIC and MBC of TCY9 against different bacterial strains.

Bacteria/Concentration (mg/mL)	50	25	12.5	6.25	3.125	1.56	0.781	Negative Control	Positive Control
*E. coli* (MIC)	−	−	−	−	+	+	+	−	+
*E. coli* (MBC)	−	−	−	+	+	+	+	−	+
*Salmonella* spp. (MIC)	−	−	−	+	+	+	+	−	+
*Salmonella* spp. (MBC)	−	−	+	+	+	+	+	−	+
*S. aureus* (MIC)	−	−	−	+	+	+	+	−	+
*S. aureus* (MBC)	−	−	+	+	+	+	+	−	+

Note: “+” indicates bacterial growth, while “−” indicates inhibition of bacterial growth for MIC and MBC. TCY3, TCY6, and TCY9 represent total flavonoid extracts obtained using three different extraction processes.

**Table 9:** Orthogonal experimental design for heat-reflux and ultrasound-assisted extraction.

Level	Ethanol Concentration	Solid-to-Liquid Ratio	Extraction Time
1	1	1	1
2	1	2	2
3	1	3	3
4	2	1	3
5	2	2	1
6	2	3	2
7	3	1	2
8	3	2	3
9	3	3	1

**Table 10:** Gradient elution program.

Time (min)	Solvent A (%)	Solvent B (%)
0	100	0
3	100	0
18.5	80	20
20	65	35
26	60	40
35	5	95
38	5	95
38.1	100	0
40	100	0

**Table 11:** Mass Spectrometry Conditions.

Parameter	Positive Ion Mode	Negative Ion Mode
Spray Voltage (V)	3800	−3200
Capillary Temperature (°C)	320	320
Aux gas heater temperature (℃)	350	350
Sheath Gas Flow Rate (Arb)	35	35
Aux gas flow rate (Arb)	8	8
S-lens RF level	50	50
Mass range (*m*/*z*)	100–1500	100–1500
Full ms resolution	60,000	60,000
MS/MS resolution	15,000	15,000
NCE/stepped NCE	10, 20, 40	10, 20, 40

## Data Availability

The datasets generated and analyzed during this study are available from the corresponding author upon reasonable request.

## Abbreviations

ABTS	2,2′-azino-bis (3-ethylbenzothiazoline-6-sulfonic acid) diammonium salt
BPCs	Base peak chromatograms
CAT	Catalase
CCK-8	Cell counting kit-8
DDA	data-dependent acquisition
DMEM	Dulbecco’s modified Eagle’s medium
DMSO	Dimethyl sulfoxide
DPPH	1,1-Diphenyl-2-picrylhydrazyl
Eq	Equation
FRAP	Ferric reducing antioxidant power
GSH-Px	Glutathione peroxidase
HESI	heated electrospray ionization
LPS	Lipopolysaccharide
MBC	Minimum bactericidal concentration
MDA	Malondialdehyde
MIC	Minimum inhibitory concentration
NO	Nitric oxide
PBS	Phosphate-buffered saline
SOD	Superoxide dismutase
